# Self‐Powered Bearing Sensing and Real‐Time Fault Diagnosis Enabled by Non‐Invasive Triboelectric Sensors and Edge AI Acceleration

**DOI:** 10.1002/advs.75373

**Published:** 2026-04-22

**Authors:** Kehui Zhu, Zhongheng Liu, Xinming Li, Jinrui Zhang, Meng Li, Yiming Guo, Lihua Han, Yanxue Wang

**Affiliations:** ^1^ School of Mechanical Electrical and Vehicle Engineering Beijing University of Civil Engineering and Architecture Beijing China; ^2^ School of Intelligence Science and Technology Beijing University of Civil Engineering and Architecture Beijing China

**Keywords:** edge acceleration, FPGA, real‐time intelligent diagnosis, self‐powered sensor, smart bearing, triboelectric nanogenerator

## Abstract

Real‐time perception of bearing operating conditions is essential for ensuring the reliable functioning of rotating machinery, yet conventional monitoring approaches that rely on complex sensor networks and external power supplies are constrained by installation space and environmental interference, hindering the realization of highly integrated, low‐power, and real‐time industrial monitoring. To address this challenge, a non‐invasive single‐electrode triboelectric bearing sensor (NSE‐TBS) is developed, which can be directly attached to the bearing surface. Based on the principle of triboelectric nanogenerators (TENG), the sensor converts mechanical energy into self‐powered condition‐sensing signals. Experimental results demonstrate that the NSE‐TBS enables stable rotational speed tracking, cage skidding detection, and fault feature extraction under various operating conditions. Furthermore, a 1D vision Transformer (1D‐ViT) diagnostic system accelerated by a field‐programmable gate array (FPGA) is implemented. Through optimized dataflow and parallel matrix multiplication engine, the model achieves an inference power consumption of only 4.59W on the FPGA, representing reductions of 4.7× and 10.4× compared with CPU and GPU implementations, respectively, with a latency as low as 0.108ms and a fault‐classification accuracy of 98%. This study achieves the integration of self‐powered sensors with edge AI acceleration, providing a new pathway for real‐time diagnostics of smart bearings.

## Graphical abstract

This study achieves the synergistic integration of self‐powered sensing and edge AI acceleration to establish a real‐time fault diagnosis system. The proposed TENG‐based self‐powered bearing sensor (NSE‐TBS) and FPGA‐accelerated edge AI framework fundamentally break through the inherent limitations of conventional monitoring systems, including complex wiring, high power consumption, and insufficient real‐time capability.

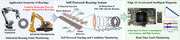

## 1 Introduction

With the continuous evolution of industrial equipment toward higher autonomy, data‐driven operation, and enhanced operational reliability, rotating machinery plays a critical supporting role in key sectors such as energy, manufacturing, and transportation [1, 2]. As the core components responsible for load transmission, power output, and structural stability in rotating machinery [3, 4], rolling bearings are highly vulnerable to causing amplified vibrations, increased friction, or even system shutdown once faults or operational anomalies occur. Such issues may lead to reduced production efficiency and, in severe cases, catastrophic equipment failure. Therefore, establishing a bearing health monitoring and fault‐warning system with high real‐time performance, continuity, and reliability is essential for ensuring the safe operation of complex industrial equipment.

Industrial intelligent diagnosis faces a persistent disconnect between front‐end sensing and back‐end computation. This structural gap hinders the simultaneous optimization of data acquisition and on‐site inference, particularly regarding physical deployment, energy supply, and architecture. In bearing health monitoring, mainstream sensing techniques such as strain monitoring [5, 6], acoustic emission detection [7, 8], and motor current analysis [9, 10] rely on complex power wiring, bulky data acquisition equipment, and remote computing resources. In addition, these sensors are typically mounted externally and cannot be positioned close to critical contact regions such as the raceway. Consequently, the signals are easily affected by noise, load variations, and structural shielding, making it difficult to obtain high‐fidelity in situ information. To address these limitations, triboelectric nanogenerators (TENGs) have emerged as a self‐powered sensing solution, enabling reliable, in situ monitoring in compact and harsh environments without external power or complex cabling [11, 12, 13, 14]. By converting frictional contact into electrical signals, TENGs inherently map the operational states of rotating bearings. This compatibility has led to the development of various TENG‐based bearing prototypes. Consequently, researchers have proposed various TENG‐based bearing prototypes. For instance, Dong et al. [15] embedded a TENG sensor within a bearing to enable defect diagnosis through its electrical output. Reference [16] developed a triboelectric sensor for monitoring the operational state of railway freight cars and established an energy self‐sufficient system for autonomous signal acquisition and transmission. However, these sensors compromise the structural integrity of the bearing, and the bearings are fabricated using 3D printing, whose material properties are not directly applicable to industrial equipment. To preserve the structural integrity of the bearing, various embedded, and external bearing sensors have been proposed in recent studies [17, 18, 19]. However, these designs often impose constraints on practical applications. For instance, Han et al. [20] integrated electrodes into the outer rings at the cost of assembly interference and reduced durability. Other approaches rely on the detachable structure of tapered roller bearings [21] or require additional auxiliary components for sensor installation [1], a modification that increases axial dimensions and limits applicability in space‐constrained environments. Moreover, most existing studies lack systematic evaluations under variable‐speed, variable‐load, and fault conditions, which are critical for reliable fault warning and condition monitoring.

On the computational side, current mainstream approaches combine the electrical output of triboelectric sensors with deep learning techniques to achieve bearing fault diagnosis. For instance, Zang et al. [22] proposed a TENG‐based dual‐peak energy harvester and achieved high‐accuracy bearing fault diagnosis by integrating a residual neural network (ResNet)18 model. Another study [23] utilized TENG signals in combination with signal decomposition methods and machine learning models to effectively identify the wear state of rolling elements. These studies demonstrate that the in situ signals provided by TENGs offer direct physical mapping and high sensitivity, providing a reliable data foundation for intelligent diagnosis of mechanical systems. However, intelligent diagnostic models such as convolutional neural networks (CNNs)[24, 25], ResNet [26, 27], and Transformers [28, 29] typically rely on high‐performance GPUs or workstations and are generally limited to offline processing. This leads to a disconnect between signal sensing and computation, hindering the realization of real‐time, low‐power diagnostic loops. Consequently, existing systems struggle to support immediate on‐site decision‐making, thereby constraining the development of truly autonomous intelligent mechanical systems.

Based on an analysis of the core contradictions in industrial scenarios, this study introduces systematic innovations in sensor design, sensing mechanisms, and intelligent computing architecture, and proposes a self‐powered intelligent bearing real‐time diagnostic solution suitable for industrial deployment. First, we designed a non‐invasive single‐electrode triboelectric bearing sensor (NSE‐TBS), which integrates energy harvesting and condition sensing within a single device. The sensor has a simple structure and can be directly attached to the outer ring and cage surface of the bearing without modifying the bearing structure, while avoiding additional complex auxiliary assembly structures. Owing to the high sensitivity of triboelectric signals to variations in contact pressure, the NSE‐TBS can accurately respond to periodic pressure disturbances induced by fault impacts. Experimental results under multiple operating conditions demonstrate that the electrical signals output by the NSE‐TBS not only reliably reflect changes in bearing rotational speed but also maintain high sensitivity to fault‐induced impacts, providing a robust raw data foundation for subsequent real‐time intelligent diagnostics.

At the real‐time intelligent diagnostic level, this study implements a 1D visual Transformer (1D‐ViT) inference architecture designed for edge deployment. We implemented a highly optimized tiled matrix multiplication engine on field‐programmable gate array (FPGA), which, combined with persistent on‐chip memory and a parallel computing architecture, significantly enhances the model's computational speed and energy efficiency, effectively overcoming the computational and power limitations typically encountered when deploying deep learning models in industrial environments. Furthermore, owing to the reconfigurable parallel architecture of the FPGA, the system can simultaneously perform real‐time signal acquisition and inference, offering clear advantages over conventional edge computing platforms such as digital signal processors (DSPs), Jetson, or Raspberry Pi. Finally, we developed an online monitoring system that deeply integrates self‐powered signal acquisition with FPGA‐based edge intelligent diagnostics. Under multiple operating conditions, the system achieved up to 98% real‐time fault diagnosis accuracy, demonstrating for the first time the feasibility of collaborative operation between triboelectric self‐powered sensing and edge intelligent inference. This provides a directly engineering‐applicable solution for the next generation of intelligent bearings with autonomous sensing and autonomous understanding capabilities. The main innovations of this work are as follows:

- Structural innovation: A non‐invasive self‐powered triboelectric sensor for industrial bearings is proposed. It preserves the mechanical integrity of the bearing, enables high‐fidelity in situ signal acquisition, and achieves autonomous energy supply. This structural design overcomes the bottlenecks that have hindered the engineering integration of conventional TENG‐based bearings sensors.
- Self‐sensing mechanism: The robust self‐sensing capability of the NSE‐TBS under multiple operating conditions is demonstrated, where its output electrical signals enable precise bearing speed measurement, cage skidding rate monitoring, and fault characteristic frequency identification, while exhibiting low load sensitivity and strong disturbance immunity.
- High‐efficiency edge intelligent computing architecture: An FPGA‐accelerated 1D‐ViT inference architecture is constructed, in which tiled matrix multiplication and data reuse strategies are employed to enable efficient model execution. Its energy efficiency is improved by factors of 59.4 and 10.8 compared with CPU and GPU implementations, respectively.
- Edge intelligent diagnostic system: The deep integration of self‐powered triboelectric sensing with edge intelligent diagnostics is achieved for the first time, resulting in an intelligent bearing system capable of real‐time operation under practical conditions. An online diagnostic accuracy of 98% is attained across multiple operating scenarios, with a diagnostic latency of only 0.108 ms.

## 2 System Design Methodology

### 2.1 Structural Design and Working Mechanism of the NSE‐TBS

**FIGURE 1:**
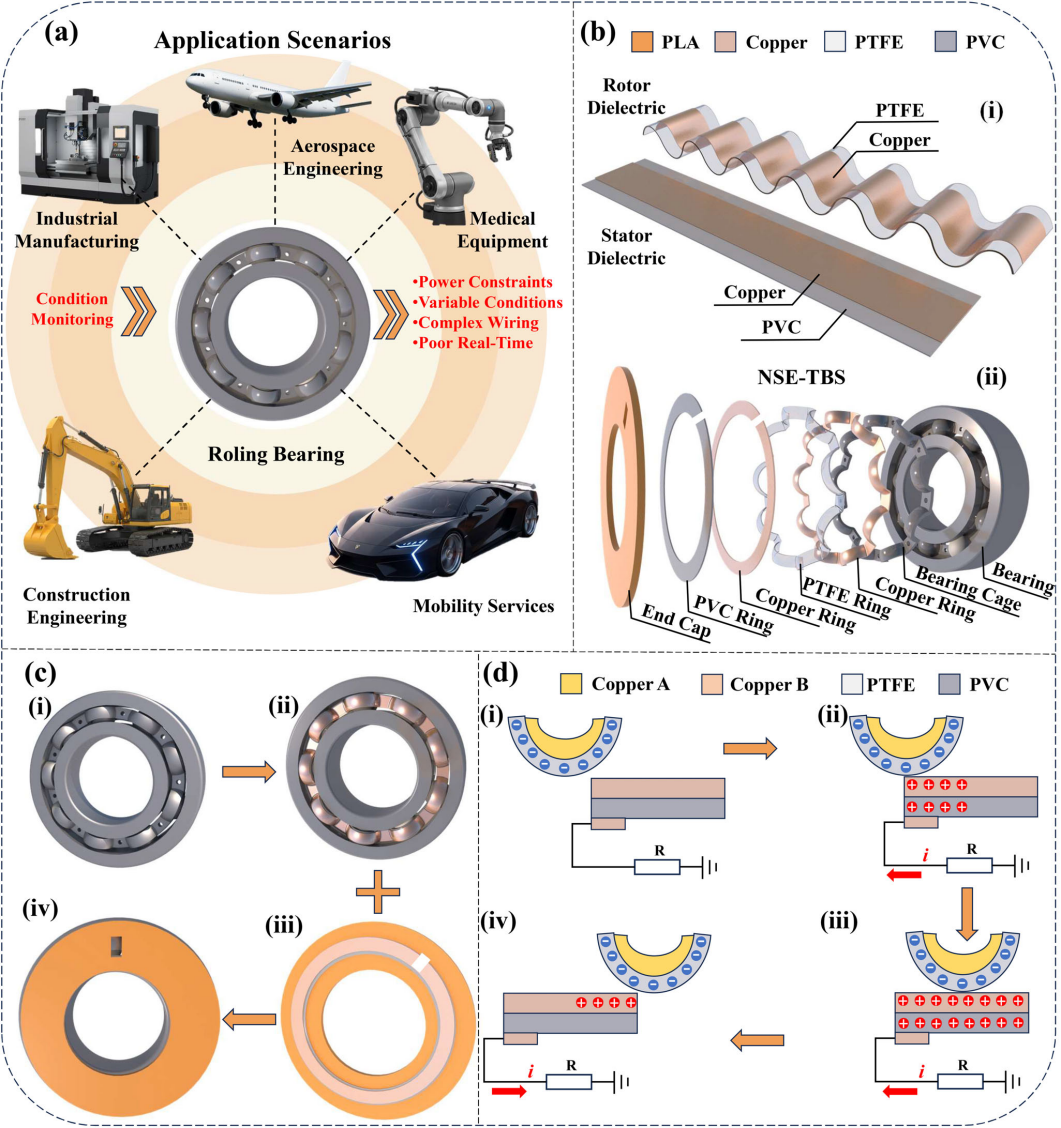
Implementation scheme of the NSE‐TBS: (a) bearing application domains, (b) sensor materials and installation exploded view, (c) installation procedure, (d) operating principle.

As illustrated in Figure 1a, bearings are extensively employed across aerospace, industrial manufacturing, transportation, and many other engineering domains. Real‐time assessment of bearing health is essential for timely adjustment of operating conditions, thereby ensuring safe and stable system operation. However, conventional monitoring approaches are constrained by challenges such as power supply complexity, wiring difficulties, and structural interference, which limit their deployment under practical working conditions and hinder effective in situ monitoring of bearing operating states.

To address these challenges, this study proposes a self‐powered state‐sensing sensor termed NSE‐TBS, which can be embedded within the bearing assembly to enable precise monitoring of its operating condition. The NSE‐TBS consists of a rotor dielectric layer and a stator dielectric layer, with the two dielectric materials shown in Figure 1 bi. The rotor dielectric layer is composed of polytetrafluoroethylene (PTFE) and copper, and is designed in a ring‐shaped wavy configuration. The stator dielectric layer is made of polyvinyl chloride (PVC) and copper, and can be fixed onto the outer surface of the bearing's outer ring. Figure 1 bii presents the exploded view of the NSE‐TBS assembled with the bearing, in which the complete structure includes the End Cap, PVC Ring, Cu Ring, PTFE Ring, Bearing Cage, and the Bearing. Unlike most existing TENG‐based bearing sensors, the proposed ring‐shaped wavy rotor dielectric structure can be directly attached to the cage surface without disassembling or modifying the bearing, thereby avoiding any impact on its original rotational characteristics. The wavy configuration maintains effective contact while significantly reducing the actual frictional area with the stator dielectric layer, thereby lowering frictional wear and extending the service life of the sensor.

The assembly sequence of the NSE‐TBS on the bearing is shown in Figure 1c. The wavy rotor dielectric layer is firmly attached to the surface of the bearing cage using pressure‐sensitive adhesive applied on the inner copper layer, ensuring complete synchronization with the cage during rotation without any relative skidding. Subsequently, the signal lead is fixed onto the PVC film surface of the stator dielectric layer to enable electrical signal output. Compared with traditional dual‐electrode TENG‐based bearing sensor structures, the single‐electrode design greatly reduces structural complexity and facilitates subsequent maintenance. The End Cap is fabricated via 3D printing and incorporates an internal boss for mounting the stator dielectric layer. The air gap between the rotor and stator can be mechanically adjusted by modifying the height of this boss. Finally, the End Cap is bonded to the bearing's outer ring, completing the integrated assembly of the NSE‐TBS. Figure S1 further illustrates the physical installation of the NSE‐TBS and provides a detailed description of the sensor fabrication and installation procedures.

As illustrated in Figure 1d, the NSE‐TBS operates as a contact‐mode sliding triboelectric structure, and its electricity generation process can be divided into four stages. In the first stage, the PTFE film on one of the rotor asperities is positioned at the signal electrode, while it remains separated from copper B embedded in the stator dielectric layer. Under this condition, the system is electrostatically balanced and no current is generated in the external circuit. In the next stage, as the cage drives the rotor electrode to approach and contact the stator electrode, frictional charge transfer occurs at the interface due to the higher electron affinity of PTFE, causing negative charges to accumulate on the PTFE surface and inducing an equal amount of positive charges on the PVC layer of the stator dielectric. The resulting potential difference drives electrons from the ground to copper B, generating a forward current to balance the potential. In the third stage, as the inner ring of the bearing continues to rotate, the two dielectric layers undergo sustained sliding contact, driving the interfacial charge separation to its maximum level and resulting in a peak potential difference in the external circuit. Finally, when the PTFE film on the rotor asperity passes over the signal electrode and gradually moves away from the stator dielectric layer, the interfacial potential reverses. Positive charges flow back from copper B to the ground terminal, giving rise to a reverse current. It is noteworthy that the rotor and stator of the NSE‐TBS are in near point contact, a configuration that endows the sensor with enhanced wear resistance.

During the entire power generation process, the NSE‐TBS continuously repeats its operation with the rotation of the bearing, producing alternating electrical signals. It is evident that the fundamental frequency of the electrical signal, $f_{v}$, is related to the speed of the rotor dielectric and can be expressed as:

(1) $$f_{v}=N_{p}\cdot f_{\text{cage}}$$

here, $N_{p}$ represents the number of electrodes, and $f_{\text{cage}}$ denotes the theoretical rotational frequency of the bearing cage. For deep groove ball bearings, the rotational frequency of the cage can be expressed as:

(2) $$f_{\text{cage}}=\frac{1}{2}f_{i}\left(1-\frac{d}{D}\cos\alpha \right)$$

where, $f_{i}$ represents the rotational frequency of the bearing inner ring, d is the diameter of the rolling element, D is the pitch circle diameter, and α denotes the contact angle. It is evident that the electrical signals output by the NSE‐TBS contain rich characteristic components, with the fundamental frequency closely related to the bearing speed and operating condition. These signals can thus be utilized to monitor both the rotational speed and the health status of the bearing.

### 2.2 FPGA‐Accelerated 1D‐ViT‐Based Intelligent Diagnostic Model

The ViT model can capture global feature dependencies through the self‐attention mechanism [30], and compared with CNNs, which have a limited receptive field and are sensitive to non‐stationarity, it demonstrates higher adaptability when processing long time‐series electrical signals. However, the classical ViT is designed for 2D image inputs, receiving sequences of 2D patches. To enable the model to directly handle 1D electrical signals acquired by the NSE‐TBS, the patch embedding layer of the ViT is modified to a 1D structure, thereby better accommodating the temporal feature patterns of the signals. Despite the targeted structural modifications, the 1D‐ViT still inherits the Transformer's dependence on dense matrix multiplications, such as Q/K/V generation, multi‐head attention fusion, and feed‐forward network computations. On conventional CPUs and GPUs, these operations require frequent access to high‐bandwidth memory, significantly increasing data movement overhead and introducing inference latency, thereby making it challenging to satisfy the stringent real‐time and energy‐efficiency requirements of industrial online diagnostics.

**FIGURE 2:**
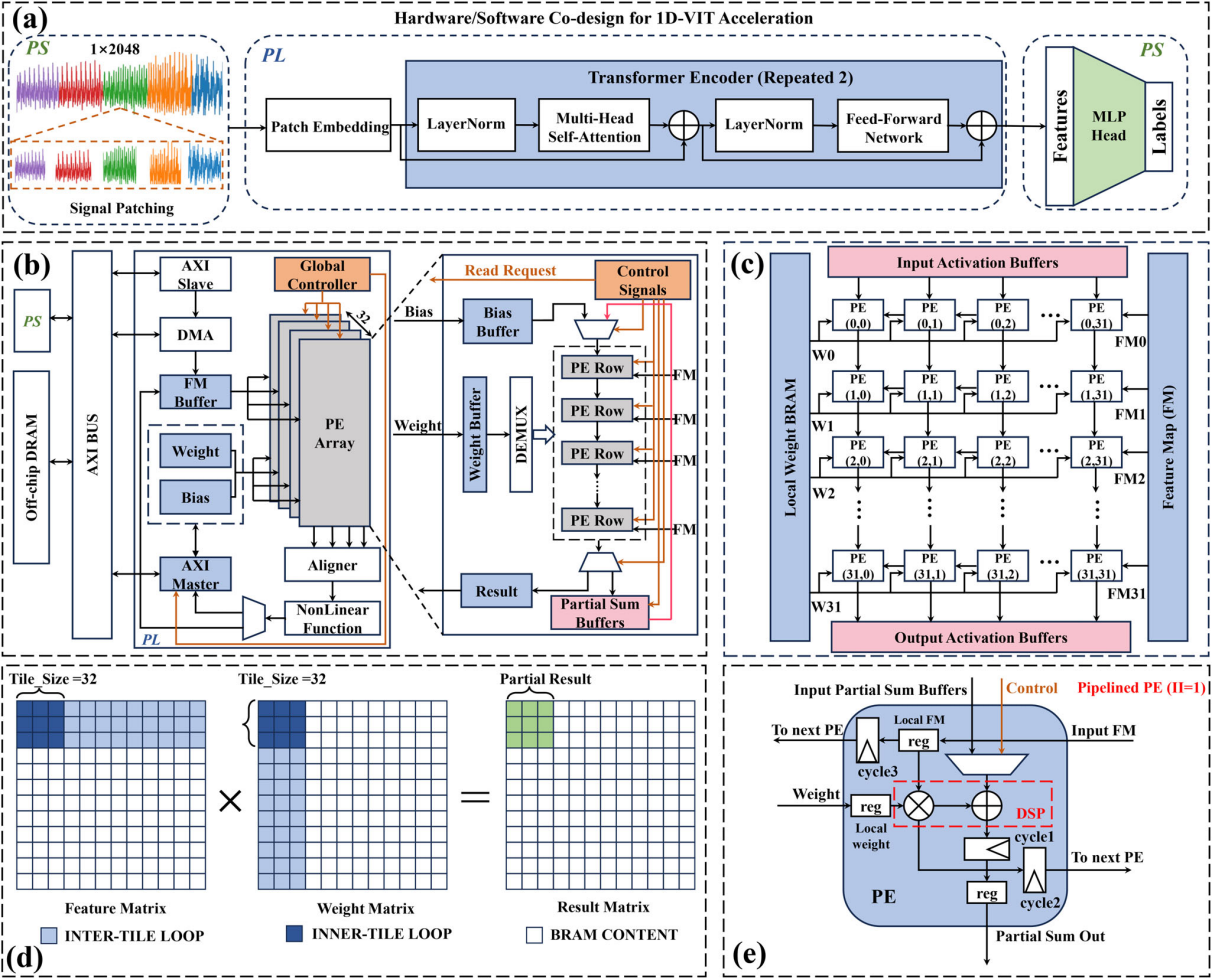
Overall accelerator framework: (a) system‐level hardware– software co‐design architecture, (b) accelerator architecture on the PL side, (c) systolic array structure, (d) matrix partitioning and computation scheme, (e) detailed structure of a PE.

To enhance the real‐time performance of intelligent diagnostics, this study proposes an FPGA‐based hardware acceleration architecture for the 1D‐ViT model. The core of this architecture lies in mapping the dense matrix multiplication operations of the 1D‐ViT onto reconfigurable hardware, enabling computational parallelism and optimized data flow, thereby significantly reducing inference latency and improving energy efficiency. Figure 2 illustrates the overall framework of the proposed accelerator and the implementation of its key components.

#### 2.2.1 System Architecture for Hardware– Software Co‐Design

The proposed accelerator is implemented on a Xilinx Zynq‐7045 FPGA, as shown in Figure 2a. The system adopts a heterogeneous architecture based on hardware– software co‐design, distributing, and coordinating the inference tasks of the 1D‐ViT between the Processing System (PS) and the Programmable Logic (PL). The PS is responsible for high‐level control logic, data transfer scheduling, and processing the final classification results. The PL, on the other hand, handles all matrix multiplication‐intensive computations, including the Patch Embedding, Multi‐Head Self‐Attention within the Transformer Encoder, the Feed‐Forward Network, and the MLP Head.

A highly parallelized general matrix multiplication (GEMM) engine is implemented on the PL to accelerate all linear layers and attention mechanism computations within the model. This GEMM architecture employs a three‐level tiled data mapping strategy combined with a systolic array structure, which significantly improves data reuse and computational throughput while effectively controlling on‐chip memory resource consumption. The overall data flow of the accelerator is illustrated in Figure 2b and is primarily composed of three subsystems:

1) Multi‐Level Memory Hierarchy and Data Flow Management. To minimize off‐chip DRAM access pressure and improve energy efficiency, data is transferred between off‐chip memory and on‐chip compute cores via high‐speed DMA modules, complemented by two types of custom caching strategies:

- Feature Matrix Persistent Cache: After partitioning the input electrical signal, the resulting feature matrix is stored in the on‐chip feature matrix Buffer. Given the high‐frequency reuse of the feature matrix in attention mechanisms (e.g., $QK^{T}$ and subsequent AV operations), the feature matrix can remain resident across multiple computation stages, eliminating frequent off‐chip DRAM accesses and thereby significantly reducing off‐chip bandwidth usage.
- Weight Streaming: Considering that weight matrices are typically large in dimension and have relatively low reuse, a streaming loading mechanism based on 256‐column blocks (or the remaining columns if fewer than 256) is employed. Each weight block is sequentially loaded into the on‐chip weight buffer via DMA, then partitioned into 32×32 tiles and directly fed into the Processing Element (PE) array, enabling efficient computation under limited BRAM resources.

2) Post‐Processing and Data Write‐Back Pipeline. The outputs of the PE array are generated in an interleaved manner with data parallelism and pipelining, and are subsequently fed into an efficient post‐processing pipeline. In this pipeline, the Aligner module first reorders and synchronizes the interleaved data to ensure the correctness of spatial positions and channel dimensions. The data then passes through the NonLinear Function module (e.g., GeLU activation, LayerNorm) before being written to the output buffer and ultimately transferred back to DRAM via the AXI bus. The entire process is orchestrated by the Global Controller, ensuring the pipelined execution of data transfer, computation, and write‐back operations.

3) Three‐Level Tiling and Systolic Array Computation Core. The core computation unit achieves efficient matrix multiplication by integrating a three‐level memory mapping (from off‐chip DRAM to on‐chip BRAM, and then to the PE Array) with an output‐stationary dataflow:

- Matrix Block Looping: The cached feature matrices and weight matrices are first partitioned into tiles of size TILE_SIZE=32 along both row and column dimensions within the Inter‐Tile Loop (as indicated by the light blue blocks in Figure 2d and sequentially loaded into BRAM. The Inner‐Tile Loop (shown as dark blue blocks in Figure 2d then feeds these tiles into the PE array for iterative computation and accumulation, with the green blocks in Figure 2d representing the computed results.
- Systolic Array Computation: The innermost loop is fully unrolled to create a 32×32 PE array, as illustrated in Figure 2c. Under the output‐stationary dataflow, the input FM enters from the top of the array, while the weights enter from the left. Partial sums are accumulated within the array and subsequently output from the bottom. At a clock frequency of 100 MHz, this design can perform up to 1,024 multiply‐accumulate (MAC) operations per clock cycle, achieving a peak performance of 102.4 G MAC s^−1^. It is noteworthy that, while maintaining a 100 MHz clock, increasing the TILE_SIZE (e.g., to 64) would pose significant challenges for FPGA routing. Selecting TILE_SIZE=32, therefor, provides an optimal trade‐off between performance and routing complexity. The internal design of each PE unit serves as the cornerstone for achieving high throughput, as shown in Figure 2e. At the core of each PE is a dedicated DSP block responsible for executing MAC operations. By employing the *#pragma HLS PIPELINE II* directive in HLS, a new MAC operation can be initiated every clock cycle, ensuring continuous, and uninterrupted data flow between PE units. Each PE receives the feature map and weights passed from the preceding unit and buffers them in dedicated register arrays. The accumulated partial sums are then forwarded to the next PE through registers, enabling efficient data access and peak throughput across the entire systolic array.

#### 2.2.2 Implementation on FPGA

The entire accelerator was developed using Vivado HLS 2023.1, with the core computation modules implemented in C++. Functional‐level simulation and cycle‐accurate verification were performed to ensure both correctness and synthesizability of the design. After verification, a synthesizable Intellectual Property (IP) core was generated and integrated into the top‐level FPGA design for final deployment. To reduce the model's memory footprint, post‐training quantization (PTQ) [31] was applied after the 1D‐ViT model had converged. The weights and biases were compressed from 32‐bit floating‐point to eight‐bit integer format, achieving approximately a 75% reduction in model storage without causing a noticeable degradation in accuracy. The system integration of the model was implemented using the Block Design flow in Vivado 2023.1, targeting the ZYNQ‐7045 SoC platform. Within the system architecture, the matrix‐multiplication IP core is interconnected with the PS side through an AXI Interconnect bus, and the entire data path adheres to the standard AXI protocol. The IP core is equipped with three AXI Master interfaces, which are responsible for reading the two input matrices and writing back the output matrix, thereby supporting high‐bandwidth data transfers efficiently. All data transfers between the PL and external DDR are managed by a DMA controller to enable batched, high‐throughput data movement. Configuration of control registers and task initiation are performed through the AXI‐Lite interface, providing flexible scheduling and precise control of acceleration tasks. After completing the Block Design construction and synthesis, the system generates the bitstream file, which is then downloaded to the FPGA for hardware deployment.

The driver software and computational logic on the PS side were implemented within a Jupyter Notebook environment. By invoking the underlying hardware acceleration modules, the system achieves collaborative software– hardware inference acceleration for the 1D‐ViT model.

## 3 Performance Analysis and Evaluation of NSE‐TBS Sensing

### 3.1 Analysis of the Output Capability of the NSE‐TBS

**FIGURE 3:**
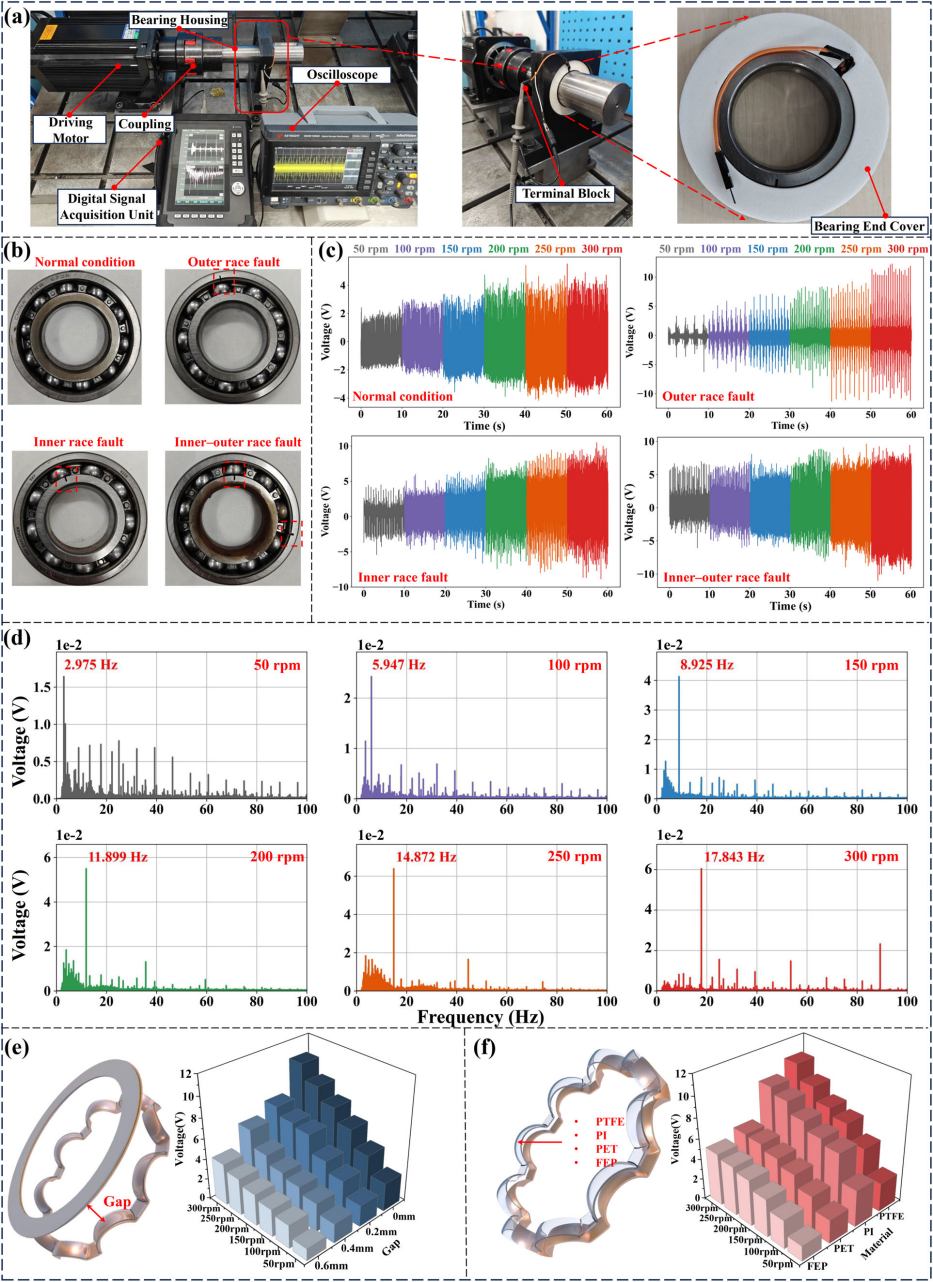
Performance evaluation of the NSE‐TBS: (a) experimental platform, (b) test bearing, (c) output voltage waveform, (d) signal spectrum, (e) gap between the rotor and stator dielectric layers, (f) rotor dielectric layer material.

This section aims to comprehensively evaluate the output characteristics of the NSE‐TBS through systematic experiments. Figure 3a illustrates the constructed experimental verification platform, which primarily consists of a drive motor, a coupling, a bearing housing, and a bearing assembly integrated with the NSE‐TBS. The testing instruments include a digital signal acquisition unit (COCO 8x) and a high‐impedance oscilloscope (KEYSIGHT DSOX1204G) to ensure accurate real‐time data visualization and acquisition.

A deep‐groove ball bearing (SKF 6208) was selected as the research subject, and four operating conditions were considered: normal condition (NC), outer race fault (OF), inner race fault (IF), and combined inner– outer race fault (IOF). The fault samples were fabricated using electrical discharge machining (EDM), producing grooves with a width of 1.8 mm and a depth of 2 mm, as illustrated in Figure 3b. The defect width accounts for only 1.1% and 0.8% of the circumferences of the inner race (approximately 157 mm) and outer race (approximately 220 mm), respectively. Owing to the minimal spatial occupancy of the defects on the raceway, the geometric continuity of the bearing operation remains largely intact, which is consistent with the physical characteristics of early faults [32]. During the experiments, the NSE‐TBS was mounted on the bearing, while an additional electrode was installed on the bearing housing as a reference ground to suppress baseline drift in the output voltage. The height of the protrusion on the end cap was adjusted so that the rotor dielectric and stator dielectric were brought into precise contact. The motor speed was set to 50– 300 rpm, corresponding to the rotational speed of the inner race. To ensure high temporal resolution in the acquisition of triboelectric signals and to fully capture the characteristic fault frequencies and their higher‐order harmonics of the 6208 bearing during operation, the sampling rate was set to 12.8 kHz. Figure 3c presents the output voltage characteristics of the NSE‐TBS under different rotational speed conditions. The results indicate that the peak voltage increases with rising rotational speed. This phenomenon is primarily attributed to the increase in the friction frequency between the rotor dielectric layer and the stator dielectric layer at higher speeds, which enhances the charge transfer effect. This observation is consistent with previous reports on the triboelectric sensing mechanism [33, 34], thereby validating the effectiveness of the NSE‐TBS under varying rotational speed conditions. Further observations reveal that, at the same rotational speed, the peak voltages of all faulty bearings are significantly higher than those of the normal bearing. This is attributed to the periodic impacts induced by the faults, which increase the contact pressure and the frequency of effective friction events, thereby enhancing the triboelectric charge transfer effect and raising the output voltage levels. To further investigate the dynamic response characteristics of the NSE‐TBS, the time‐domain signals of a healthy bearing at rotational speeds ranging from 50 to 300 rpm were analyzed using the Fast Fourier Transform (FFT), with the results presented in Figure 3d. Based on the structural parameters of the SKF 6208 bearing (d = 12.3 mm, D = 60 mm, α = 0, number of rolling elements = 9), the number of pole pairs corresponding to the rotor dielectric electrode $N_{p}$ is = 9. The measured dominant frequency agrees well with the theoretical fundamental frequency $f_{v}$ derived from Equations (1) and (2), indicating that the voltage signal generated by the NSE‐TBS can accurately reflect the bearing's rotational speed. As the speed increases from 50 to 300 rpm, the spectrum exhibits a relatively high signal‐to‐noise ratio (SNR), characterized by a clear and sharp fundamental peak, while the noise floor remains consistently low. Moreover, the presence of abundant harmonic components in the spectrum reflects the inherently non‐sinusoidal, impulsive nature of the triboelectric sensing signal, indicating that the NSE‐TBS possesses excellent temporal resolution and is capable of precisely capturing transient charge variations during the bearing's rotational process. These high‐fidelity signal characteristics provide a rich and reliable source of features for subsequent high‐precision bearing fault diagnosis. It is worth noting that the slight deviation between the measured dominant frequency and the theoretical value typically indicates the presence of cage slip, which may arise from factors such as localized defects, slight misalignment, or load fluctuations. At low rotational speeds (e.g., 50– 100 rpm), a small number of spurious components observed in the low‐frequency region reflect nonlinear disturbances under low dynamic energy levels. As the rotational speed increases, the energy distribution of the spectral peaks becomes more concentrated, indicating enhanced dynamic stability of the system under high‐speed operating conditions. This suggests that the output signals of the NSE‐TBS are sensitive to variations in rotational speed and the motion state of the bearing cage, providing critical information for enabling self‐sensing capabilities of the bearing.

In industrial applications, deep‐groove ball bearings often experience axial displacement due to installation errors, thermal expansion, and uneven load distribution, which can create a small gap between the rotor dielectric and the stator dielectric. This structural gap alters the contact charge density and the electric field coupling strength between the two dielectric layers, thereby affecting the energy conversion efficiency of the NSE‐TBS. To quantitatively assess the effect of the gap on output performance, the height of the protrusion on the end cap was precisely adjusted using 3D printing, varying the dielectric layer gap in 0.2 mm increments. Experiments were conducted under the outer race fault condition, as shown in Figure 3e. The results indicate that the output voltage of the NSE‐TBS decreases with increasing gap, which is attributed to the reduction in contact area and the weakened electric field coupling, thereby suppressing the charge transfer process [35]. Therefore, to ensure optimal sensing performance in engineering applications, the protrusion height should be properly designed or adjusted to control the dielectric gap. In addition, the rotor dielectric material has a significant impact on the output performance of the NSE‐TBS. Four materials with the same thickness (0.1 mm) were compared in this study: PTFE, PI, PET, and FEP. Experiments were conducted on bearings with an outer race fault, as shown in Figure 3f. The results indicate that PTFE achieved the highest output voltage at most rotational speeds, although at 50 rpm and 100 rpm its output was slightly lower than that of PI. Nevertheless, PTFE offers significant advantages in terms of corrosion resistance, self‐lubrication, and long‐term stability [36], making it more suitable as a rotor dielectric material. Considering both output performance and engineering reliability, PTFE was ultimately selected as the optimal material for the rotor dielectric layer.

### 3.2 Speed and Skidding Rate Monitoring Capability of the NSE‐TBS

**FIGURE 4:**
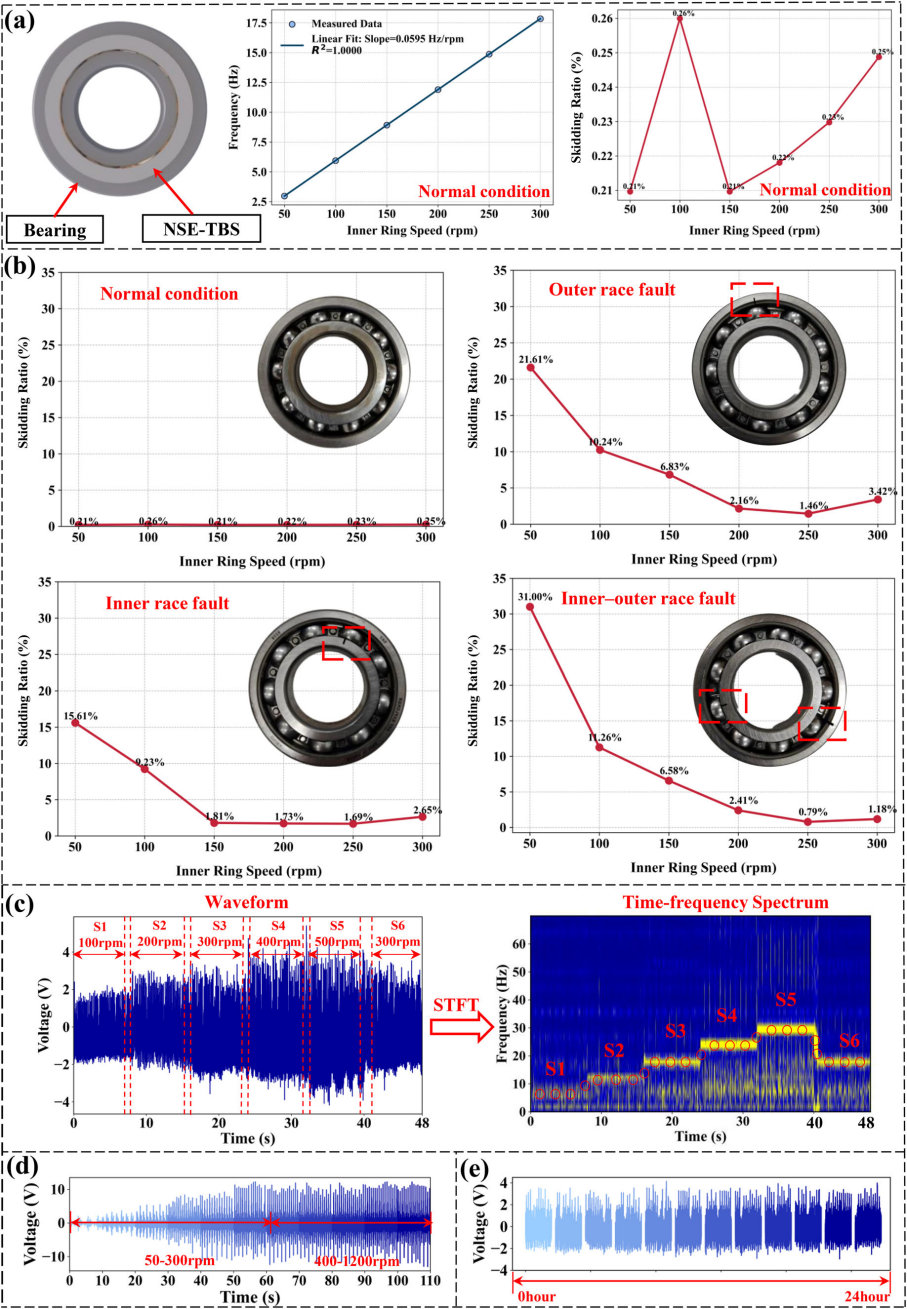
Application diagram of the NSE‐TBS: (a) speed tracking, (b) skidding rate analysis, (c) variable‐speed tracking capability, (d) high‐speed performance validation, (e) output voltage under long‐term continuous operation.

Further experiments verified the ability of the NSE‐TBS to sense bearing rotational speed under various operating conditions. As shown in Figure 4a, under the healthy bearing condition, when the rotational speed increased from 50 to 300 rpm, the fundamental frequency of the NSE‐TBS output signal exhibited a highly linear relationship with the rotational speed, with a linear fit coefficient of determination $R^{2}=1.0000$. This result demonstrates the ultra‐high accuracy of the NSE‐TBS in speed detection, providing a reliable basis for subsequent experimental analyses. Deviations of the measured cage rotational frequency from the theoretical value can be regarded as indications of cage skidding [37]. Severe cage skidding may lead to lubricant film rupture, which in turn accelerates bearing component wear and fatigue failure [38, 39]. These effects not only significantly reduce bearing service life but also compromise system operational reliability, highlighting the importance of accurate monitoring of cage skidding. According to Equation (1), the cage rotational frequency exhibits a ninefold ($N_{p}$ = 9) mapping relationship with the fundamental frequency of the triboelectric signal, the measured fundamental frequency ${\hat{f}}_{v}$ can be extracted from the FFT of the acquired triboelectric signal. By comparing ${\hat{f}}_{v}$ with the theoretical fundamental frequency $f_{v}$, the cage skidding ratio $S_{c}$ can be quantitatively determined, as given by:

(3) $$S_{c}=\frac{\left|{\hat{f}}_{v}-f_{v}\right|}{{\hat{f}}_{v}}$$

The measured fundamental frequency, theoretical fundamental frequency, their deviation, and the calculated cage skidding rates for four different bearing conditions at rotational speeds ranging from 50 to 300 rpm are summarized in Table S1. The cage skidding ratio under the normal bearing condition is first illustrated in Figure 4a. It can be observed that the bearing cage exhibits varying degrees of skidding across the entire rotational speed range, which is typically attributed to clearance variations during assembly, installation errors, and unstable lubrication conditions. At a rotational speed of 100 rpm, the skidding rate reaches a maximum of 0.26%, yet overall it remains at a relatively low level. To evaluate the sensitivity of the NSE‐TBS under different bearing fault conditions, the cage skidding characteristics of IF, OF, and IOF bearings were investigated. The experimental results, as presented in Table S1 and Figure 4b, indicate that the introduction of faults significantly amplifies the degree of skidding. The direct perception of skidding behavior by the NSE‐TBS stems from the frequency response characteristics of its signal. Mechanical skidding of the bearing cage induces a rotational lag relative to its theoretical motion, which is synchronously encoded as a reduction in the pulse frequency of the triboelectric sensing signal. In the frequency domain, this phenomenon is manifested as a negative deviation of the fundamental frequency relative to the theoretical rotational frequency. Specifically, at 50 rpm, the maximum skidding rates for the IF, OF, and IOF conditions reach 21.61%, 15.61%, and 31.00%, respectively, which are significantly higher than those of the healthy bearing. This quantified deviation between the measured fundamental frequency and the theoretical fundamental frequency (see the frequency deviation term in Table S1) establishes a direct link between cage skidding behavior and the triboelectric signal output, demonstrating that the NSE‐TBS can directly perceive variations in the internal motion state of the bearing cage without the need for additional sensors. In fact, bearing skidding and bearing faults are mutually coupled. On one hand, cage skidding can cause local contacts to transition from rolling friction to sliding friction, increasing the friction coefficient and accelerating wear, which leads to cumulative bearing damage. On the other hand, faulty bearings often experience temperature rise, lubricant film breakdown, and periodic impacts during operation, which further destabilize the instantaneous motion of the cage and exacerbate skidding. It is noteworthy that in the high‐speed region, due to the enhanced centrifugal force, the rolling elements are pressed more stably against the raceways, resulting in a reduction of skidding behavior. In Figure 4b, the cage skidding rates of all three faulty bearings are noticeably reduced at high rotational speeds, which may cause the faulty bearings to appear similar to healthy bearings under such conditions. However, this does not imply that the fault risk is eliminated, as early‐stage damage continues to accumulate. Therefore, the timely detection of bearing damage at low speeds is of critical importance. The experimental results demonstrate that the NSE‐TBS can clearly capture the more pronounced cage skidding characteristics of faulty bearings compared to healthy bearings in the low‐speed range, validating its high sensitivity to early faults and providing a crucial basis for distinguishing early‐stage bearing health conditions.

The aforementioned experiments were conducted under constant rotational speed, whereas in industrial systems, machinery typically operates with variable speeds. Therefore, the speed‐tracking capability of the NSE‐TBS under variable‐speed conditions was further evaluated. Experiments were carried out under healthy bearing conditions, with the bearing rotational speed dynamically varied by adjusting the input speed of the driving shaft. As shown in Figure 4c, the time‐domain waveforms of the NSE‐TBS output exhibit pronounced differences across different speed stages. Further analysis using short‐time Fourier transform (STFT) [40] reveals that the dominant frequency in the time– frequency spectra accurately reflects the temporal evolution of the rotational speed (the regions of speed variation are highlighted by red circles in the figure). This result indicate that the NSE‐TBS can reliably and in real time sense bearing speed variations under variable‐speed conditions, making it suitable for more complex operating scenarios in industrial environments. Finally, the variation of the NSE‐TBS output peak voltage at higher rotational speeds was investigated. To clearly capture the peak voltage changes, experiments were conducted on bearings with an outer race fault, with the rotational speed adjusted in two stages: the first stage from 50 to 300 rpm with 50 rpm increments, and the second stage from 400 to 1200 rpm with 200 rpm increments. Each speed was maintained for 8 s, while the sensor voltage data were continuously recorded. As shown in Figure 4d, the peak output voltage of the NSE‐TBS gradually increases with rotational speed. In the high‐speed region (400−1200 rpm), the triboelectric charge density reaches its maximum, and the variation in transferable charge becomes stable, resulting in a steady peak voltage of approximately 10 V. This indicates that the NSE‐TBS maintains reliable output performance under high‐speed operating conditions. To verify the long‐term operational reliability of the sensor, a 24‐h continuous operation experiment was conducted. At a rotational speed of 100 rpm, the output electrical signals of the NSE‐TBS were collected every 2 h, as shown in Figure 4e. The results indicate that no significant attenuation in the peak‐to‐peak voltage of the NSE‐TBS output signal is observed under prolonged operation, demonstrating excellent stability. This stability is mainly attributed to the structural design of the wavy rotor dielectric layer, which supports the reliable long‐term operation of the NSE‐TBS. Overall, the experimental results in this section systematically validate the output performance and speed‐sensing capability of the NSE‐TBS under constant‐speed, variable‐speed, and high‐speed conditions. The sensor demonstrates excellent dynamic adaptability, providing a solid foundation for bearing condition monitoring and intelligent fault diagnosis in complex industrial scenarios.

## 4 Sensing Performance and Assessment of NSE‐TBS under Complex Conditions

**FIGURE 5:**
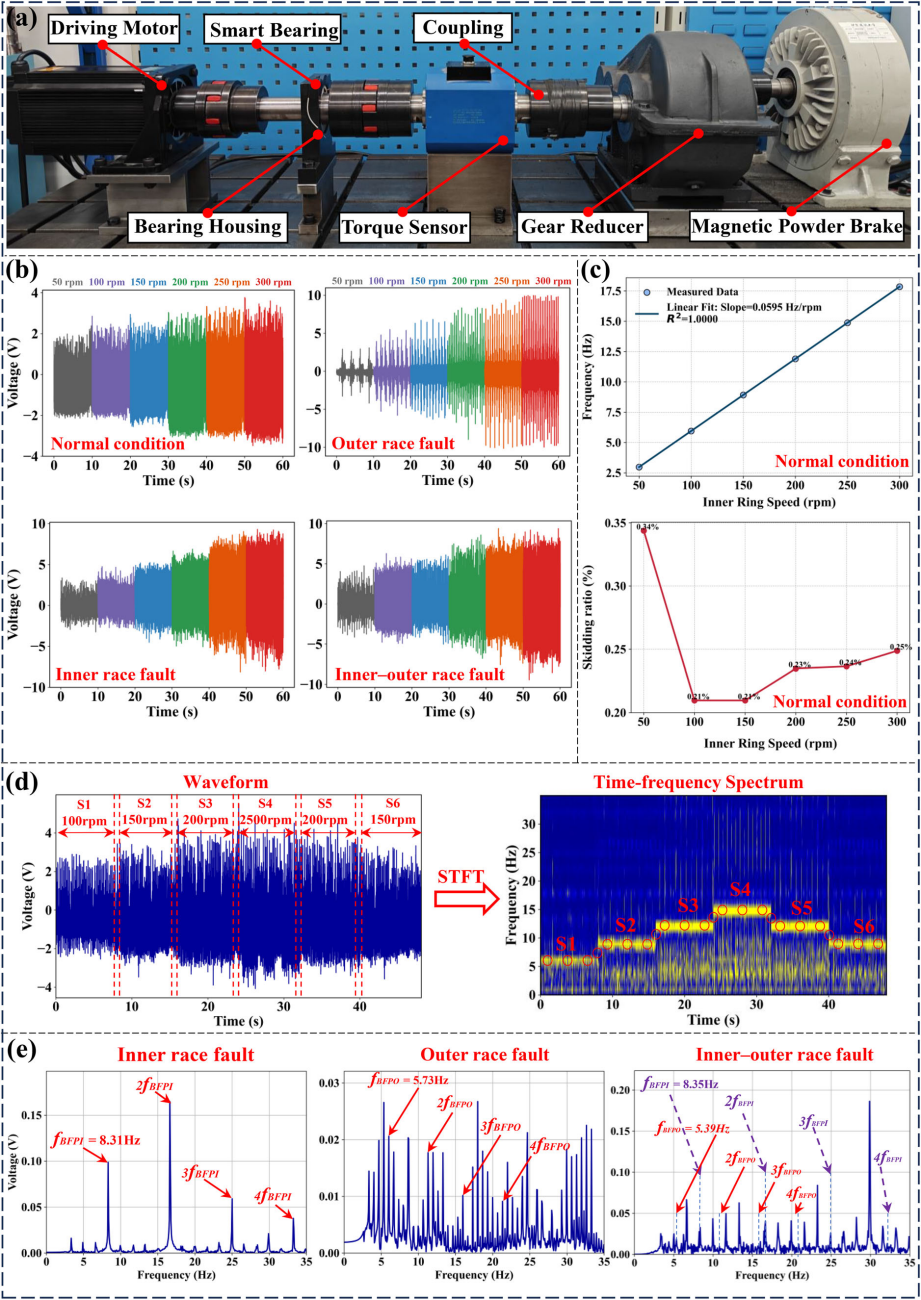
Performance evaluation of the NSE‐TBS under complex operating conditions: (a) est platform, (b) voltage signals, (c) speed fitting and skidding rate monitoring, (d) variable‐speed tracking, (e) validation of fault monitoring capability.

Previous experiments were primarily conducted under no‐load or relatively simple operating conditions. However, during real‐world operation, mechanical systems are often subjected to unbalanced forces, coupled vibrations, and environmental disturbances, which may induce fluctuations in sensing signals and degrade output accuracy. Therefore, it is necessary to further investigate and validate the output stability of the NSE‐TBS under load conditions, as well as its potential capability for fault monitoring. To this end, a load‐bearing experimental platform was constructed, as shown in Figure 5a. The platform consists of a test motor, coupling, an intelligent bearing unit integrated with the NSE‐TBS, bearing housing, torque sensor, gear reducer, and magnetic powder brake. During operation, the system generates multi‐source vibration coupling and random mechanical impacts, thereby realistically reproducing load variations and noise disturbances commonly encountered in industrial environments. This creates a reliable experimental basis for evaluating the sensor's performance.

The signal acquisition Video S1. Figure 5b presents the electrical output signals of the NSE‐TBS under four bearing health conditions. Overall, the peak voltage increases with rotational speed, reaching up to approximately 10 V. Under fault conditions, pronounced impulsive features emerge in the signal. Notably, the influence of load on the NSE‐TBS output is minimal, and its performance stability can be attributed to its non‐intrusive structural design, which prevents direct exposure to radial forces and effectively mitigates mechanical stress‐induced signal disturbances. This characteristic stands in sharp contrast to most TENG‐based sensors that are required to sustain mechanical loads [41, 42, 43, 44, 45]. Figure 5c illustrates the rotational speed sensing performance of the NSE‐TBS and the variation in cage skidding rate under healthy bearing conditions. The results show an excellent linear correlation between the dominant frequency of the output signal and the bearing speed, with a fitting coefficient of $R^{2}=01.0000$, indicating that the NSE‐TBS retains exceptionally high speed sensing accuracy even in complex load‐bearing environments. Although load‐induced operation introduces additional friction and disturbances, leading to a slight increase in cage skidding rate (up to approximately 0.34%), the overall level remains low, demonstrating the stability of the operating system. Moreover, to evaluate the potential impact of the NSE‐TBS on the mechanical efficiency of the bearing system, a comparative torque measurement experiment was conducted. As shown in Figure S2 and Table S2, the additional torque loss introduced by the NSE‐TBS is only about 1%, confirming that the proposed sensing scheme has a negligible effect on the mechanical efficiency of the system. The speed‐tracking capability of the NSE‐TBS was further evaluated by dynamically adjusting the motor speed. Specifically, the motor speed was gradually increased from 100 to 150 rpm, 200, and 250 rpm, and then decreased back to 200 and 150 rpm. As shown in Figure 5d, the NSE‐TBS output signals clearly reflect the changes in rotational speed in both the time‐domain waveforms and the time– frequency spectrograms. The dominant frequency components in the time– frequency spectrogram (highlighted with red circles) closely correspond to the actual speed variations, demonstrating that the NSE‐TBS can accurately capture dynamic changes in bearing speed even under multi‐source interference.

In typical industrial systems, surrounding mechanical components generate complex background vibrations and structural resonances, and such random or impulsive interferences can significantly compromise the detectability of bearing fault signals, particularly at low rotational speeds. For instance, in heavy‐load equipment such as low‐speed turbines, the energy excited by bearing faults is often lower than the system background noise, causing the characteristic impact signals to be easily masked [46]. Therefore, in addition to high‐precision speed sensing, the NSE‐TBS must also be capable of accurately extracting fault features under complex vibration environments and low‐speed conditions. The typical bearing fault characteristic frequencies are as follows:

(4) $${\hat{f}}_{BPFI}=\frac{1}{2}Nf_{i}\left(1+\frac{d}{D}\cos\alpha \right)$$

(5) $${\hat{f}}_{BPFO}=\frac{1}{2}Nf_{i}\left(1-\frac{d}{D}\cos\alpha \right)$$

here, ${\hat{f}}_{BPFI}$ and ${\hat{f}}_{BPFO}$ represent the theoretical bearing fault frequencies for the inner and outer rings, respectively. The experiment was conducted at a motor speed of 100 rpm, and based on the parameters of a 6028 bearing, the calculated frequencies were ${\hat{f}}_{BPFI}$ = 9.0375 Hz and ${\hat{f}}_{BPFO}$ = 5.9625 Hz. To accurately extract the fault frequencies and their harmonic components from the signals, a 2−35 Hz band‐pass filter was first applied to the raw electrical signals to suppress low‐frequency drift and high‐frequency noise, followed by FFT analysis to obtain the frequency spectra. Figure 5e presents the spectra for three bearing fault conditions, in which both the fundamental fault frequencies and their harmonics are clearly discernible. Under the IF condition, the measured $f_{BPFI}$ was 8.31 Hz, exhibiting an 8.05% deviation from the theoretical value. This discrepancy is primarily attributed to cage skidding, which results in an actual rotational speed lower than the theoretical one, thereby reducing the fault excitation frequency. Similarly, under the OF and IOF conditions, the fundamental fault frequencies also exhibited varying degrees of reduction, with the outer ring fault showing a 3.90% deviation, and the inner and outer ring frequencies in the IOF condition deviating by 7.61% and 9.60%, respectively. Despite these minor deviations from the theoretical values, the spectra maintain clear harmonic structures, indicating that the signals remain highly resolvable. The above frequency‐domain analysis demonstrates that the NSE‐TBS can effectively identify different types of bearing faults and exhibits strong robustness against fault frequency deviations. Considering that industrial bearings typically operate under complex lubrication conditions, experiments were conducted under oil‐rich lubrication to evaluate the sensing robustness of the NSE‐TBS. The corresponding results are presented in Figure S3, demonstrating that the sensor can still achieve high‐precision rotational speed detection and cage skidding analysis under lubricated conditions. Taken together, the multi‐condition experimental results validate that the NSE‐TBS maintains excellent anti‐interference capability and high sensitivity under both steady‐state and dynamic operating conditions, providing a reliable foundation for subsequent real‐time monitoring and intelligent diagnostics.

## 5 Real‐time Intelligent Bearing Diagnosis Enabled by FPGA Acceleration

**FIGURE 6:**
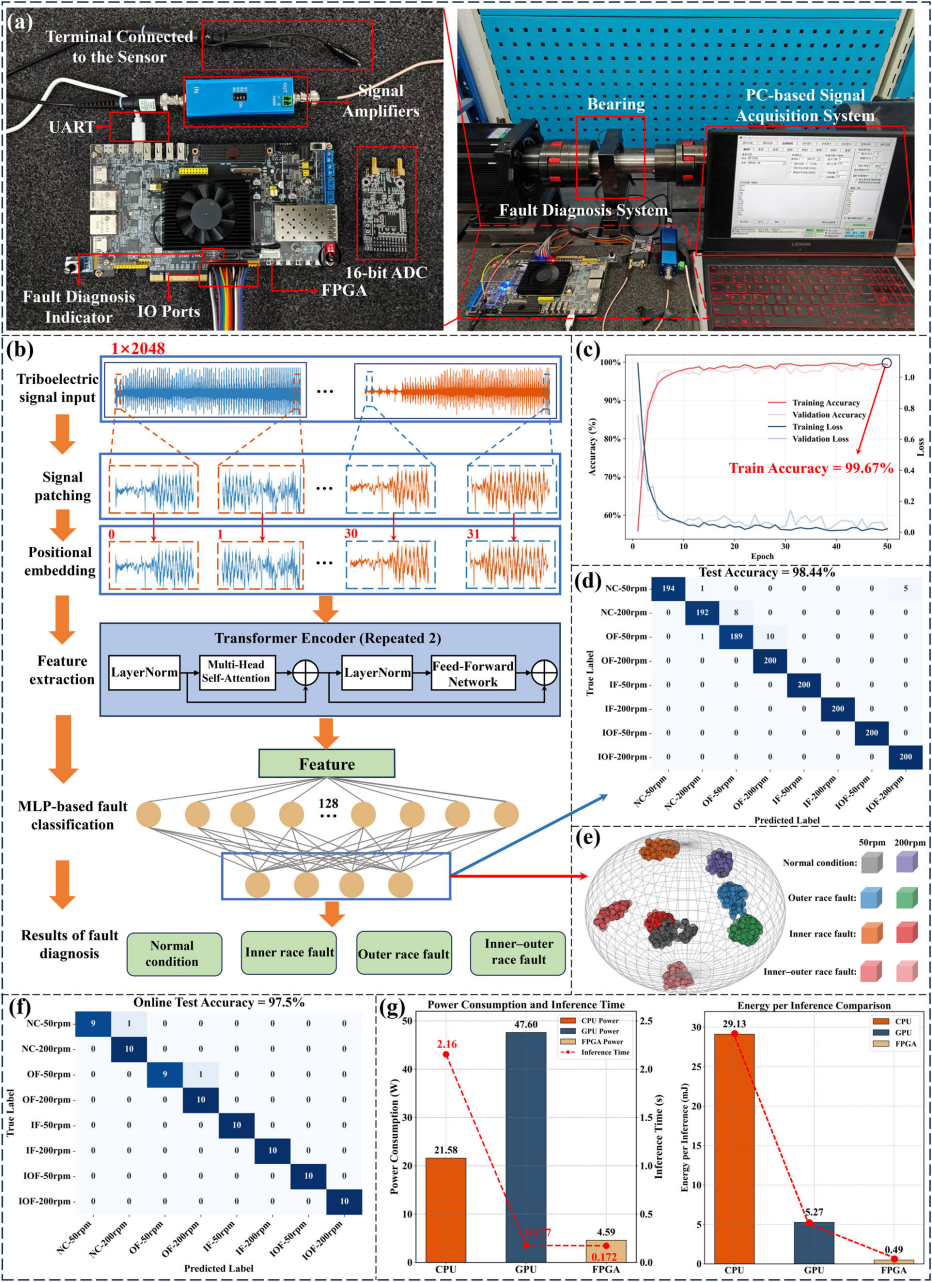
Online diagnostic evaluation: (a) real‐time diagnostic system, (b) model inference workflow, (c) training curves, (d) test accuracy, (e) t‐SNE visualization, (f) online diagnostic accuracy, (g) accelerator performance.

In real industrial environments, bearings are subjected to load fluctuations, variations in lubrication, and environmental disturbances, where minor defects can rapidly develop into severe failures such as spalling, accelerated wear, or seizure, potentially causing abrupt performance degradation of critical rotating components. Therefore, real‐time acquisition of bearing operational signals and on‐edge diagnostics are crucial for the early identification of potential risks and ensuring safe and reliable equipment operation. In this study, an FPGA‐accelerated 1D‐ViT online bearing fault monitoring system was developed. As shown in Figure 6a, the electrical signals output by the NSE‐TBS are first fed into a preamplifier through a terminal block, which adjusts the signal amplitude to the ±5 V range. The analog signals are then digitized via a 16‐bit analog‐to‐digital converter (ADC) and transmitted to the FPGA for end‐to‐end intelligent diagnostics. The diagnostic results are displayed in real time through three LED indicators.

The experiments were conducted using bearings representing four fault types under two rotational speed conditions (50 and 200 rpm), resulting in eight operating conditions. The NSE‐TBS output signals were acquired via FPGA at a sampling rate of 12.8 kHz. The collected data were transmitted to a host computer via UART and stored for model training and validation. For each condition, signals were continuously recorded for 128 s and then divided into training, validation, and test sets in a 2:1:1 ratio according to chronological order. Each sample consisted of 2048 data points, yielding a total of 3200 training samples and 1600 validation and test samples.

The diagnostic model was first constructed and trained using Python 3.10 and PyTorch 1.9.1 on a Windows 11 platform equipped with an NVIDIA GTX 1650 GPU and an Intel i5‐9000 CPU. Detailed model architecture and hyperparameter definitions are provided in Tables S3 and S4. As illustrated in Figure 6b, the diagnostic workflow begins by segmenting the raw electrical signals into fixed‐length patches to capture local features while reducing computational complexity. Positional embedding is then applied to each patch to preserve spatial information, preventing the permutation invariance of the Transformer attention mechanism from causing positional ambiguity. The encoded sequence is subsequently fed into the Transformer Encoder and processed twice to enhance feature representation capability. The resulting feature vectors are passed to an MLP‐based classifier, where a softmax layer outputs the probability distribution of the four bearing conditions (NC, IF, OF, and IOF). The loss curve and training accuracy of the model are shown in Figure 6c. After 50 training epochs, the model gradually converged, achieving a training accuracy of 99.67%. The model parameters were then frozen, and its performance was evaluated on the test set. As illustrated in the confusion matrix in Figure 6d, the overall test accuracy reached 98.44%. Only the OF‐50 rpm condition exhibited a slightly lower classification accuracy, which nevertheless remained as high as 94.5%. Furthermore, a t‐SNE nonlinear dimensionality reduction algorithm was applied to visualize the high‐dimensional features extracted by the model. As shown in Figure 6e, different fault categories exhibit well‐separated clusters in the feature space, while samples of the same class are compactly grouped. This indicates that the model possesses robust feature extraction capability.

On this basis, FPGA‐based online testing was conducted to verify real‐time diagnostic performance. The experimental conditions were consistent with those used during model training. Once the motor speed stabilized, the FPGA was activated for online diagnosis, and the diagnostic results were recorded synchronously according to the fault indicator LEDs. Each operating condition was tested ten times, and the resulting confusion matrix is presented in Figure 6f. The online testing achieved an accuracy of 97.5%, closely matching the performance obtained on the PC platform. Under low‐speed operating conditions (50 rpm), misclassification occurred between healthy bearings and those with outer‐race defects. This phenomenon is likely attributed to random cage skidding at low rotational speeds, which weakens the periodicity of impact signatures and introduces additional noise. Consequently, the distinguishability of fault characteristics is reduced, leading to deviation in diagnostic results.

Further experiments compared the inference latency and power consumption of FPGA, CPU, and GPU implementations. Based on the test dataset, power consumption during CPU and GPU inference was monitored using AIDA64, whereas the FPGA power consumption was estimated using Vivado 2023.2. Each experiment was repeated ten times, and the average performance was reported. As shown in Figure 6g, in terms of diagnostic real‐time performance, the FPGA requires only 0.172 s to process 1,600 test samples, corresponding to a single‐sample inference latency of merely 0.108 ms, which is 12.6 times faster than the CPU and also surpasses the GPU. Regarding power consumption, the FPGA reduces energy usage by factors of 4.7 and 10.4 compared with the CPU and GPU, respectively. Such low latency and power consumption can be attributed to the superior design of our accelerator. Moreover, the energy consumption per inference is an important metric for evaluating whether edge AI acceleration [47, 48, 49] and industrial edge inspection [50, 51] can operate continuously under low power. It is calculated as E=P×t/n, where P denotes power consumption, t represents inference latency, and n is the number of inference samples. The results indicate that a single inference on the FPGA consumes only 0.49 mJ, corresponding to energy efficiency improvements of 59.4× and 10.8× compared with the CPU and GPU, respectively, further demonstrating the high energy‐efficiency advantage of the accelerator.

Finally, to ensure the reliable operation of the accelerator on the FPGA, it is necessary to further evaluate its on‐chip resource utilization. Table 1 presents the main resource consumption of the matrix multiplication module. Look‐Up Tables (LUTs) and Flip‐Flops (FFs), which are used to implement combinational and sequential logic, respectively, exhibit utilization rates of 43.96% and 31.95%. The DSPs used for implementing MAC operations exhibit a utilization rate of 57%, indicating that the matrix multiplication module makes full use of the available multipliers while still maintaining ample margin. The core of the proposed deployment scheme is a general‐purpose matrix multiplication module implemented on FPGA, where TILE_SIZE defines the granularity of matrix partitioning and determines the parallelism of the PE array. To optimize hardware performance, Table S5 provides a detailed analysis of the impact of different TILE_SIZE configurations on FPGA hardware performance. Overall, the utilization of all resources remains within reasonable ranges, which helps improve timing closure and reduce placement and routing pressure, thereby ensuring the stable operation of the accelerator on the FPGA. In summary, this section presents an online bearing fault monitoring system built upon the FPGA accelerator, which leverages the electrical signals output by the NSE‐TBS to achieve high‐precision, low‐latency, and low‐power real‐time fault diagnosis under multiple operating conditions. The integration of the self‐powered sensor with the FPGA‐based monitoring system provides a novel solution for real‐time bearing fault diagnosis in complex industrial scenarios.

**TABLE 1:** FPGA Resource Utilization Summary.

Resource	Utilization	Available	Utilization [%]
LUT	96094	218600	43.96%
LUTRAM	294	70400	0.42%
FF	138114	437200	31.59%
BRAM	65.50	545	12.02%
DSP	513	900	57.00%
BUFG	3	32	9.38%

## 6 Algorithm Robustness Evaluation Combined Speed Conditions and Hardware Performance Comparison

**FIGURE 7:**
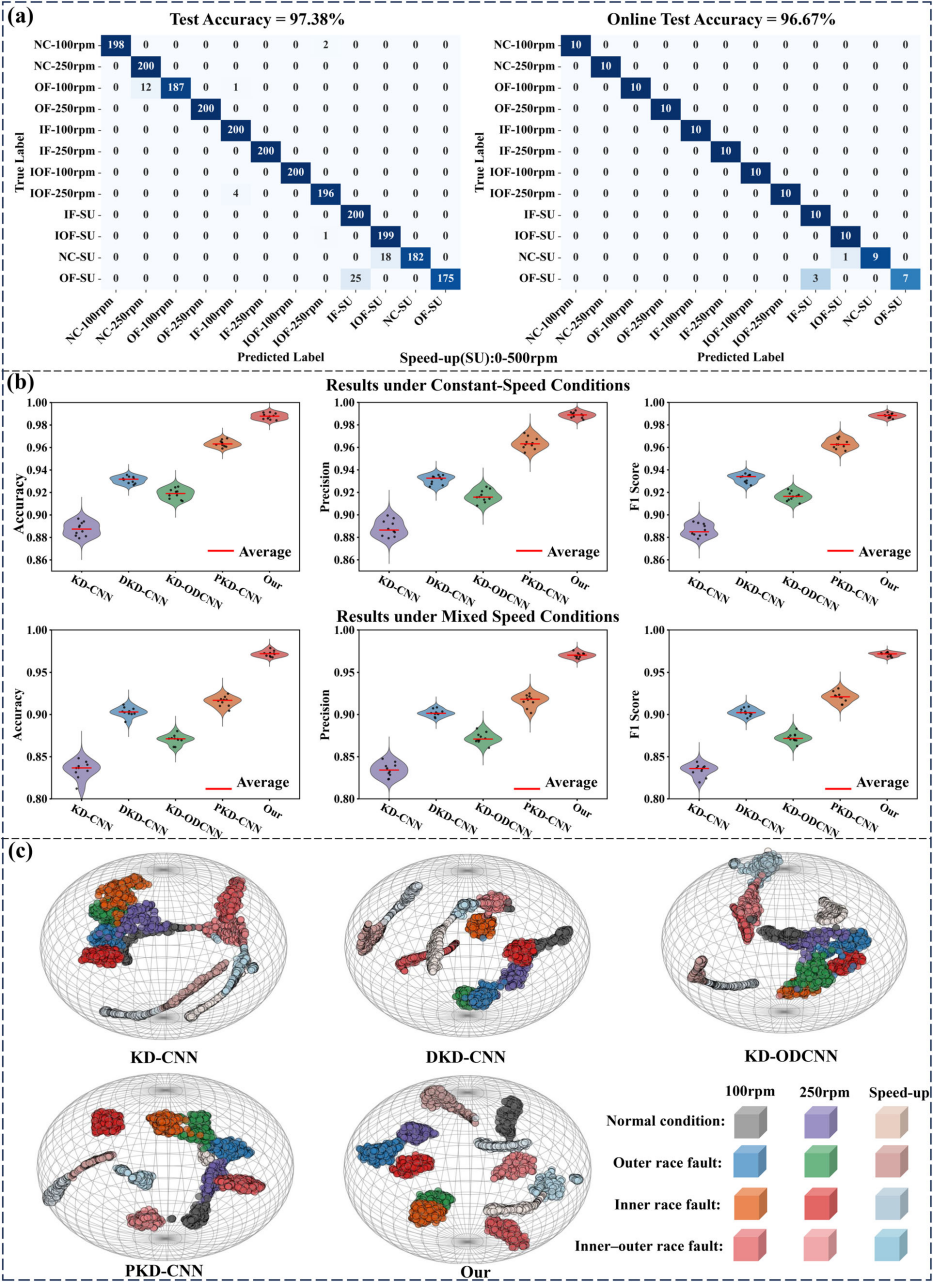
Performance comparison of different intelligent diagnostic methods: (a) comparison of diagnostic accuracy between offline diagnosis and FPGA‐Based online diagnosis, (b) performance comparison of different models, (c) t‐SNE visualization of different models under mixed speed conditions.

The aforementioned results demonstrate that the proposed monitoring system achieves satisfactory diagnostic performance under single operating conditions. To further evaluate the model's generalization capability under more complex scenarios, experiments were conducted under variable‐speed conditions based on four typical bearing fault states. In the experiments, the motor speed ranged from 0 to 500 rpm with an acceleration time of 10 s, and the signal sampling frequency was set to 12.8 kHz. Signal acquisition started at motor startup and continued throughout the entire acceleration process. In terms of dataset construction, fault data corresponding to four bearing conditions were collected under two constant‐speed conditions (100 and 250 rpm), resulting in eight categories in total. In addition, four categories of variable‐speed data were incorporated to construct a dataset covering both constant‐speed and variable‐speed conditions. The raw signals were first segmented using a sliding window with a window length of 2048 and a step size of 28 to generate a sufficient number of samples. The samples were then divided into training, validation, and test sets in chronological order with a ratio of 2:1:1. Ultimately, 800 samples were constructed for each operating condition. Based on the aforementioned hybrid speed‐condition dataset, the test results of the model are presented in Figure 7a, achieving a classification accuracy of 97.38%. Furthermore, to evaluate the stability of the system for online diagnosis, each operating condition was independently tested ten times. The average online diagnostic accuracy reached 96.67%, indicating that the proposed monitoring system maintains good robustness under complex non‐stationary conditions. A total of four misclassifications occurred during all tests, all of which appeared under variable‐speed conditions. Among them, three cases misclassified OF as IF, while one case involved misclassification between other categories. This phenomenon can be mainly attributed to the rapid variations in dynamic load and rotor imbalance forces during acceleration, which induce pronounced non‐stationary modulation in the fault‐induced impulsive signals. As a result, the time‐domain feature distributions of different fault types partially overlap, increasing the difficulty of discrimination for the 1D‐ViT model.

To further evaluate the overall performance of the proposed accelerator, several representative CNN– FPGA acceleration methods in the field of bearing fault diagnosis were selected as baselines for comparison, including a knowledge distillation (KD)‐based CNN model (KD‐CNN) [52], a single‐layer CNN model based on decoupled knowledge distillation (DKD‐CNN) [53], a KD‐trained binarized CNN model (KD‐ODCNN) [54], and a CNN model based on progressive knowledge distillation (PKD‐CNN) [55]. The experimental data were collected from triboelectric signals under steady‐state load conditions at 100 rpm and 250 rpm, as well as under dynamic acceleration conditions. The performance evaluation metrics include accuracy, precision, and F1‐score.

To ensure the statistical reliability of the results, each experiment was independently repeated ten times under different random seeds, and the final results are reported in the form of mean ± standard deviation. The hyperparameter settings of the compared models were kept consistent with those in the original studies, with necessary adjustments made according to the number of classes in the classification task. The experimental results are presented in Table 2. It can be observed that the proposed model achieves the best performance across all three metrics. It is worth noting that, to accommodate the hardware resource constraints of the FPGA, all baseline methods employ compressed CNN architectures. Although knowledge distillation can alleviate the performance degradation caused by model compression to some extent, CNNs primarily rely on local receptive fields for feature extraction, which limits their ability to model long‐range dependencies under complex operating conditions, thereby constraining further improvements in diagnostic performance.

**TABLE 2:** Diagnostic Accuracy, Precision, and F1‐Score (%) of Different Models under Two Operating Conditions.

Model	Constant speed condition			Mixed speed condition		
	Accuracy	Precision	F1‐Score	Accuracy	Precision	F1‐Score
KD‐CNN	88.75±0.61	88.81±0.72	88.64±0.77	83.47±1.02	83.44±0.82	83.39±0.73
DKD‐CNN	93.02±0.43	93.12±0.39	93.15±0.53	90.31±0.55	90.19±0.40	90.27±0.43
KD‐ODCNN	91.88±0.76	91.66±0.53	91.64±0.64	87.04±0.56	87.21±0.65	87.25±0.55
PKD‐CNN	96.35±0.79	96.38±0.54	96.32±0.63	91.55±0.46	91.63±0.59	91.59±0.70
**Our**	98.78±0.27	98.87±0.26	98.82±0.31	97.17±0.34	97.12±0.22	97.10±0.23

To analyze the stability of each method across repeated experiments, Figure 7b presents the violin plot distributions of different diagnostic metrics. It can be observed that the proposed method exhibits higher and more compact distributions across all three evaluation metrics, indicating smaller performance fluctuations, better convergence consistency, and the absence of significant outliers. In contrast, KD‐CNN, which employs only basic knowledge distillation for CNN training, shows lower and wider distributions, suggesting more dispersed predictions and poorer stability. Although DKD‐CNN and PKD‐CNN improve the average performance through enhanced KD strategies, their diagnostic results still exhibit noticeable fluctuations under combined speed conditions due to limited model capacity. To further explain the above performance differences from a feature representation perspective, Figure 7c presents t‐SNE‐based feature visualizations. The results show that lightweight CNN models struggle to form clear decision boundaries between different fault categories. For example, in the PKD‐CNN visualization, significant overlap among feature clusters of different classes can be observed, indicating limited inter‐class separability in the feature space. In contrast, the proposed model forms more compact and well‐separated clusters, demonstrating its ability to better preserve discriminative information related to fault categories under complex operating conditions.

In terms of hardware efficiency, Table 3 summarizes the performance of the proposed accelerator in comparison with the baseline methods. The proposed accelerator achieves a normalized diagnostic latency of 54.00 μs per 1k data and an EPI of 0.49 mJ, both of which are slightly higher than those of Refs.[53] and [55]. This difference mainly arises from the higher computational complexity and matrix operation density of the 1D‐ViT model compared with CNN‐based models. However, from a system‐level perspective, the proposed method still maintains relatively low latency and energy consumption, meeting the requirements of real‐time fault diagnosis under high sampling rates as well as low‐power operation. Although the methods in Refs.[53] and [55] achieve lower energy consumption, their diagnostic accuracy is comparatively limited. Benefiting from an efficient parallel computing architecture, the proposed accelerator attains a computational throughput of 102.4 GOPS, significantly outperforming these methods. Its energy efficiency reaches 22.3 GOPS/W, which is 23× and 112× higher, respectively. These results indicate that the proposed accelerator can deliver strong computational capability under a limited power budget, achieving a well‐balanced trade‐off among diagnostic accuracy, latency, and energy consumption.

**TABLE 3:** Hardware Performance Comparison with Other FPGA Accelerators.

Paper	Bits	Algorithm	FPGA device	Clock	Throughput	Norm. Latency	EPI	Energy Efficiency
				[MHz]	[GOPS]	[μs k^−1^]	[mJ]	[GOPS/W]
[52]	8‐bit	CNN	ZYNQ‐7020	50	1.97	336.75	0.690	0.96
[53]	16‐bit	CNN	XC7K325T	100	1.18	43.82	0.029	1.76
[54]	2‐bit	CNN	ZYNQ‐7010	100	3.29	418.97	0.960	1.42
[55]	8‐bit	CNN	ZYNQ‐7045	150	0.38	36.68	0.069	0.20
Our	8‐bit	1D‐ViT	ZYNQ‐7045	100	102.40	54.00	0.490	22.30

Moreover, the overall system performance depends not only on the optimization of the algorithmic model and hardware architecture, but is also influenced by the quality of the sensed signals at the front end. To validate the advantages of the NSE‐TBS in signal representation and diagnostic applications, a quantitative comparison was further conducted between the NSE‐TBS and a conventional vibration accelerometer. The two types of sensors were evaluated in terms of signal feature extraction capability and intelligent diagnostic performance, with the corresponding results shown in Figure S4. The results demonstrate that the NSE‐TBS can provide input signals with a higher SNR, thereby offering a more favorable foundation for improving the performance of downstream models.

## 7 Conclusion

This study proposes a scheme for bearing self‐sensing and real‐time fault diagnosis by deeply integrating the self‐powered NSE‐TBS sensor with an edge AI accelerator. The proposed NSE‐TBS adopts a non‐intrusive design, in which the wavy rotor dielectric layer is directly attached to the bearing cage, while the stator dielectric layer is fixed to the bearing outer ring via the end cap. This design preserves the mechanical integrity of the bearing and demonstrates excellent versatility. Experiments under multiple operating conditions indicate that increasing the rotational speed and reducing the design clearance both enhance the output voltage amplitude of the NSE‐TBS. Moreover, the electrical signals generated by the sensor contain rich feature information, enabling high‐precision bearing speed measurement, cage skidding analysis, and fault frequency identification without an external power supply. Moreover, to enable real‐time intelligent bearing fault diagnosis, a highly optimized matrix multiplication module was implemented on the FPGA to accelerate the 1D‐ViT diagnostic model, and an online monitoring system compatible with the NSE‐TBS was constructed. The test results show that the system achieves 98% online diagnostic accuracy under various operating conditions. Meanwhile, the power consumption of FPGA inference is reduced by factors of 4.7 and 10.4 compared with the CPU and GPU, respectively, and the energy consumption per inference is only 0.49 mJ, corresponding to energy efficiency improvements of 59.4× and 10.8× over the CPU and GPU. The integration of the NSE‐TBS with the FPGA‐accelerated diagnostic model demonstrates the simultaneous advantages of low power consumption, low latency, and high accuracy, providing a novel approach for real‐time intelligent bearing diagnosis under complex operating conditions and laying the foundation for the industrial application of self‐powered sensors and intelligent diagnostic technologies.

## Conflicts of Interest

The authors declare no conflicts of interest.

## Supporting information


**Supporting File 1**: advs75373‐sup‐0001‐SuppMat.pdf.

**Supporting File 2**: advs75373‐sup‐0002‐VideoS1.mp4.

## Data Availability

The data that support the findings of this study are available from the corresponding author upon reasonable request.
