# Supplementary material for: Self‐Powered Bearing Sensing and Real‐Time Fault Diagnosis Enabled by Non‐Invasive Triboelectric Sensors and Edge AI Acceleration
Source: Adv Sci (Weinh). 2026 Apr 22;13(40):e75373. doi: 10.1002/advs.75373 (PMC13335464; doi:10.1002/advs.75373)
Supplement: Supplementary file 1 — Supporting File 1: advs75373‐sup‐0001‐SuppMat.pdf. [file ADVS-13-e75373-s002.pdf]

Supporting Information

Self-Powered Bearing Sensing and Real-Time Fault Diagnosis Enabled by Non-Invasive Triboelectric Sensors and Edge AI Acceleration

*Kehui Zhu, Zhongheng Liu, Xinming Li, Jinrui Zhang, Meng Li, Yiming Guo, Lihua Han, Yanxue Wang**

Dr. Kehui Zhu, Dr. Zhongheng Liu, Dr. Xinming Li, Dr. Jinrui Zhang, Dr. Meng Li, Yiming Guo, Prof. Yanxue Wang*
School of Mechanical, Electrical and Vehicle Engineering, Beijing University of Civil Engineering and Architecture, Beijing 100044, China
Email Address: wangyanxue@bucea.edu.cn
Lihua Han
School of Intelligence Science and Technology, Beijing University of Civil Engineering and Architecture, Beijing 102616, China

Note1: Fabrication and Installation of NSE-TBS.

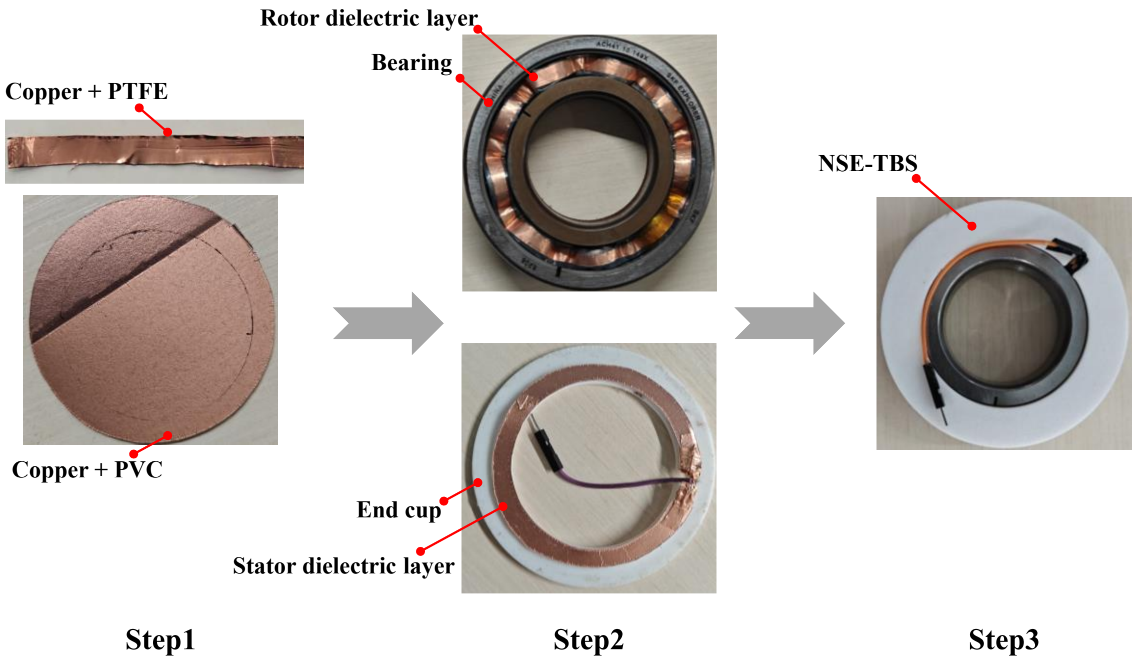

Figure S1: Fabrication and installation process of the NSE-TBS

The NSE-TBS features a simple structure, facilitating ease of fabrication and compatibility with most types of bearings. The fabrication and installation procedures are illustrated in Figure S1, and the detailed steps are as follows:：

Step1: Copper foil, PTFE film, and PVC film, each with a thickness of 0.1 mm, are selected as functional materials. A strip-shaped copper foil with dimensions of 4 mm × 250 mm is bonded to a PTFE film to construct the subsequent wavy rotor dielectric layer. Meanwhile, an annular copper foil with a diameter of 80 mm (corresponding to the outer diameter of a 6208 bearing) is bonded to a PVC film for the fabrication of the stator dielectric layer.

Step2: The PTFE–copper composite structure is bent into a wavy configuration and embedded onto the bearing cage, serving as the rotor dielectric layer. The wavy structure enables good conformity with the cage geometry. Subsequently, the PVC–copper composite is cut into an annular structure with an outer diameter of 64 mm and an inner diameter of 56 mm to form the stator dielectric layer, ensuring that it can be fitted into the gap between the inner and outer rings of the bearing. Furthermore, a bearing end cover is fabricated using 3D printing technology (inner diameter: 53 mm; outer diameter: 80 mm).

A raised platform with a height of 1.2 mm is designed on the inner side of the end cover to secure the stator dielectric layer. An opening with dimensions of 3 mm × 5 mm is created at the inner diameter position of the end cover for electrode lead-out. The stator dielectric layer (with the PVC film facing inward) is then bonded onto the platform surface and cut according to the position of the opening. Finally, the electrode is attached to the surface of the PVC film and fixed with copper foil, thereby completing the integration of the stator dielectric layer with the end cover.

Step3: After the rotor dielectric layer is installed, the end cover integrated with the stator dielectric layer is mounted onto the outer ring of the bearing, and the electrode is routed through the opening in the end cover, thereby completing the overall assembly of the NSE-TBS.

Note2: Analysis of Fault Characteristic Frequencies and Skidding Rates.

Table S1: Comparison between theoretical and measured fault characteristic frequencies and analysis of skidding rates under diverse operational conditions.

Rotational Speed (rpm)	Theoretical Frequency (Hz)	Bearing Condition	Measured Frequency (Hz)	Frequency Deviation (Hz)	Skidding Rate (%)
50	2.981	Normal	2.975	0.006	0.21
		Outer race fault	2.337	0.644	21.61
		Inner race fault	2.516	0.465	15.61
		Inner–outer race fault	2.057	0.924	31.00
100	5.963	Normal	5.947	0.016	0.26
		Outer race fault	5.352	0.611	10.24
		Inner race fault	5.412	0.551	9.23
		Inner–outer race fault	5.291	0.672	11.26
150	8.944	Normal	8.925	0.019	0.21
		Outer race fault	8.333	0.611	6.83
		Inner race fault	8.782	0.162	1.81
		Inner–outer race fault	8.355	0.589	6.58
200	11.925	Normal	11.899	0.026	0.22
		Outer race fault	11.667	0.258	2.16
		Inner race fault	11.719	0.206	1.73
		Inner–outer race fault	11.638	0.287	2.41
250	14.906	Normal	14.872	0.034	0.23
		Outer race fault	14.689	0.217	1.46
		Inner race fault	14.655	0.251	1.69
		Inner–outer race fault	14.789	0.117	0.79
300	17.888	Normal	17.843	0.045	0.25
		Outer race fault	17.276	0.612	3.42
		Inner race fault	17.414	0.474	2.65
		Inner–outer race fault	17.676	0.212	1.18

Note3: Analysis of System Torque Variation before and after NSE-TBS Installation.

To investigate whether the NSE-TBS introduces additional frictional losses and affects mechanical efficiency, comparative experiments were conducted using a healthy bearing. The experiments were carried out under identical rotational speeds (50–300 rpm) and loading conditions. A torque sensor (sensitivity $K = 300 N \cdot m/V$) was used to measure the average

torque-response voltage signal of the system before and after the installation of the NSE-TBS. The experimental setup is shown in Figure S2a. The torque-related voltage signals were acquired using a digital data acquisition unit (COCO 8X). Once the rotational speed reached a steady state, signals were continuously recorded for 15 s at a sampling rate of 12.8 kHz.

Under different rotational speeds (50, 100, 150, 200, 250, and 300 rpm), time windows with a length of 500 samples were randomly selected from the torque signals collected before and after the installation of the NSE-TBS. These segments were then superimposed for comparison, as illustrated in Figure S2b.

It can be observed that the signals before sensor installation (red) and after installation (blue) exhibit good consistency in both phase and amplitude, indicating that the introduction of the NSE-TBS induces only slight perturbations to the transient torque response of the system. On this basis, the average torque-related voltage under the two conditions was further calculated, and the torque increment introduced by the NSE-TBS was defined. Based on the measured voltage variation, the additional frictional power consumption ΔP introduced by the sensor can be expressed as:

$$\Delta P = \frac{2\Pi \cdot n \cdot (\Delta V \cdot K)}{60} \tag{S1}$$

where, n denotes the rotational speed (rpm), ΔV represents the average voltage increment before and after the installation of the sensor, and K is the sensor sensitivity coefficient.

All experimental data are summarized in Table S2. The results show that, compared with the inherent transmission resistance of the system (including bearing friction and shaft resistance), the introduction of the NSE-TBS leads to only a slight torque increment of 0.97%–1.54%. Across all tested rotational speeds, this slight increment remains relatively stable, indicating that a consistent and predictable mechanical interaction is maintained between the sensor and the bearing. Furthermore, according to Equation S1, the additional power consumption induced by the sensor is only 0.31 W at 50 rpm; even at a higher rotational speed of 300 rpm, this value increases to merely 3.768W. These results demonstrate that the proposed sensor introduces negligible disturbance to the original dynamic behavior of the bearing (e.g., cage motion state) and has a minimal impact on the overall mechanical efficiency.

Table S2. Torque-Induced Voltage Comparison and Quantification of Additional Power Consumption with and without NSE-TBS under Different Rotational Speeds

	50rpm	100rpm	150rpm	200rpm	250rpm	300rpm
With NSE-TBS	0.0132	0.0254	0.0279	0.0313	0.0353	0.0368
Without NSE-TBS	0.0130	0.0251	0.0275	0.0310	0.0348	0.0364
Voltage Increment	0.0002	0.0003	0.0004	0.0003	0.0005	0.0004
Voltage Increase Ratio	1.54%	1.20%	1.45%	0.97%	1.44%	1.10%
Power Consumption Increase	0.314W	0.942W	1.884W	1.884W	3.925W	3.768W

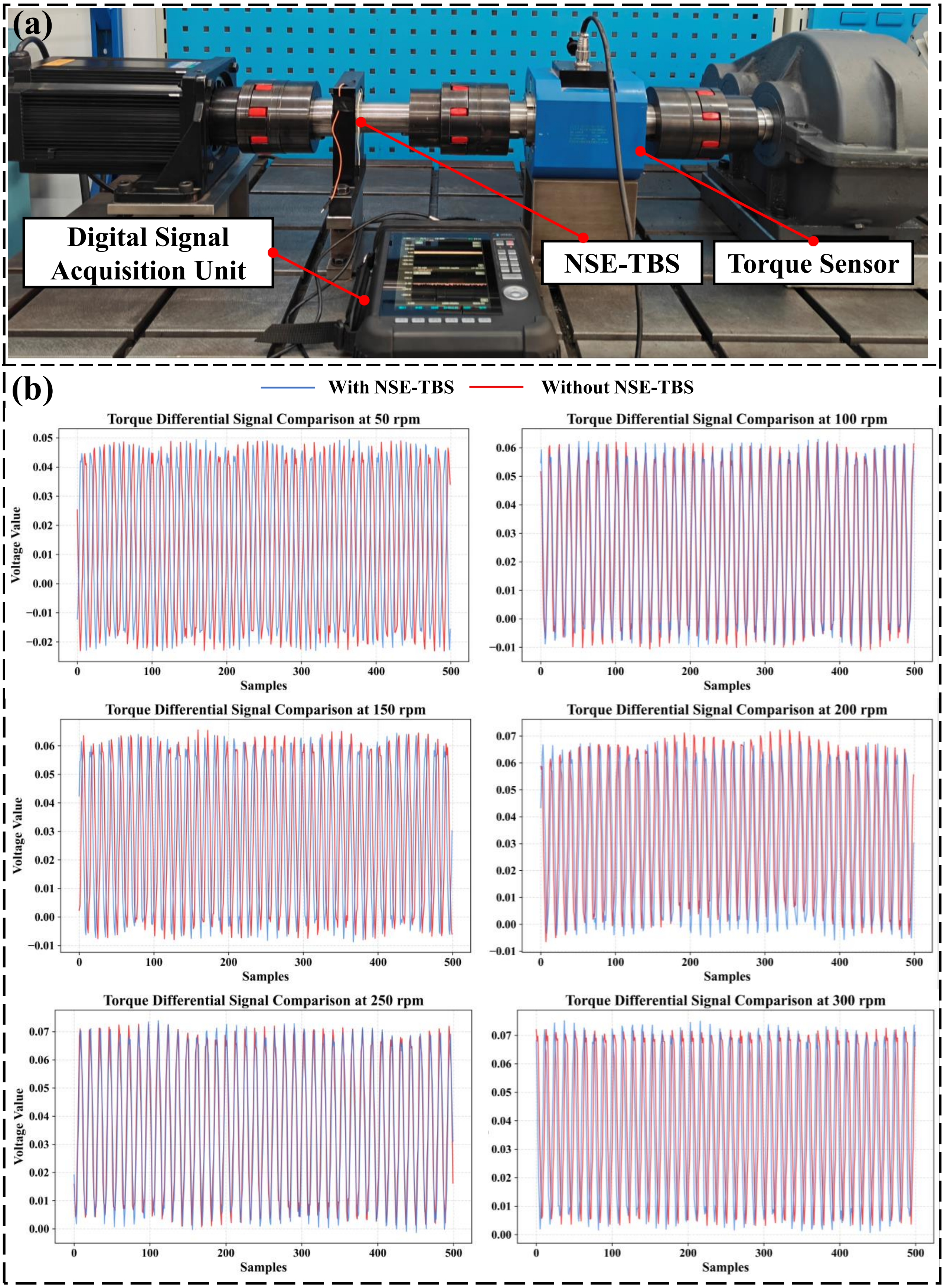

Figure S2: Comparison of torque response before and after NSE-TBS installation: a) experimental setup, b) voltage signal comparison.

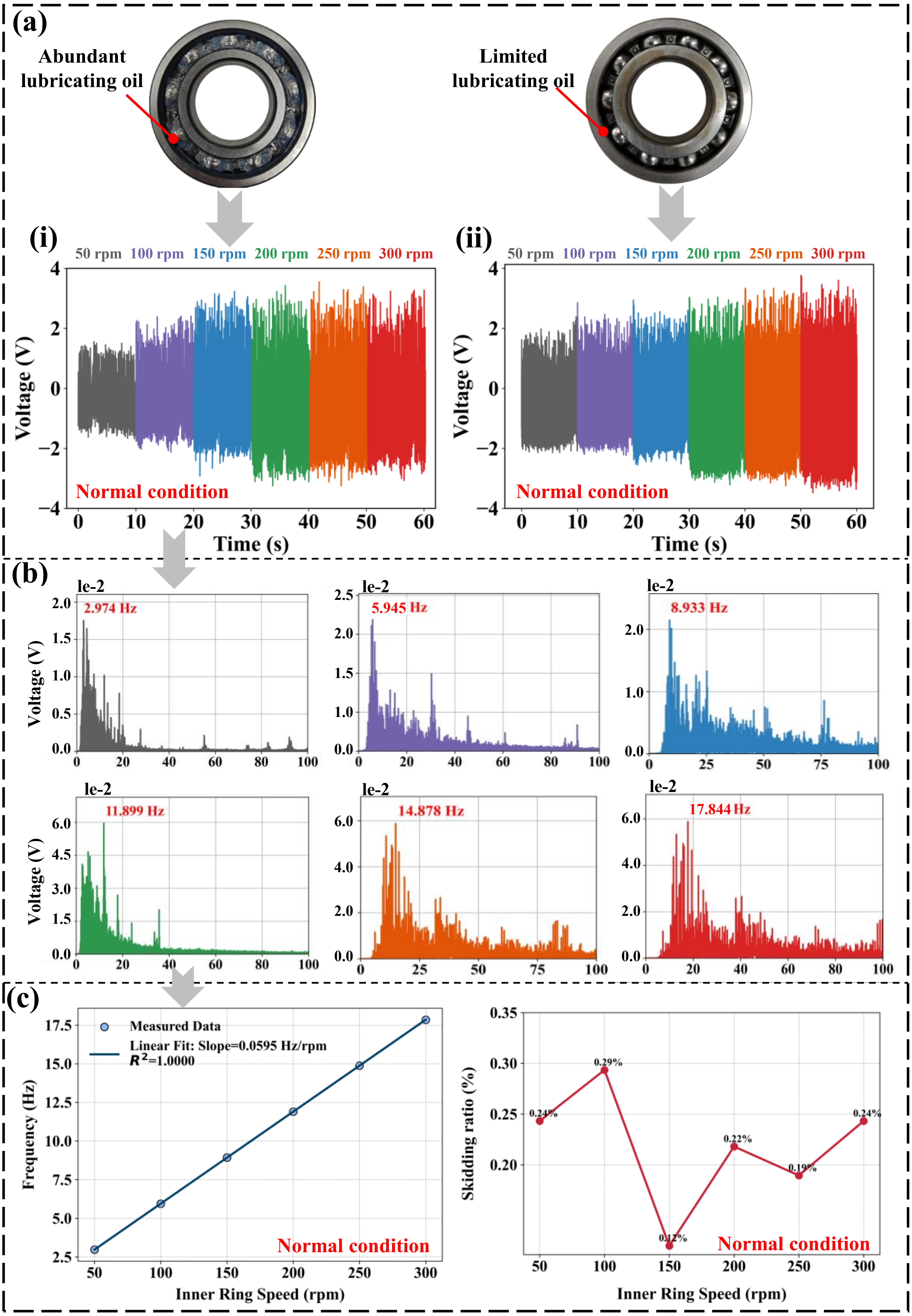

Figure S3: Output performance analysis of the NSE-TBS under lubricated conditions: a) voltage time-domain signal, b) frequency spectrum derived from the voltage time-domain signal, c) rotational speed detection results and cage skidding rate analysis.

Note4: Performance Analysis of NSE-TBS under Lubricated Conditions.

To evaluate the effect of lubrication on the output performance of the NSE-TBS, experiments were conducted on a fully lubricated bearing under load conditions. During the experiments, the motor speed was set from 50 to 300 rpm, and the electrical output signals of the NSE-TBS are shown in Figure S3a (i). Compared with the results obtained from the bearing

with a small amount of lubricant (Figure S3a (ii)), no significant variation in the peak voltage of the output signal is observed. Furthermore, the raw signals under different rotational speeds were processed using the Fast Fourier Transform (FFT) to extract the characteristic frequencies, as shown in Figure S2b. Based on the extracted frequencies, the actual rotational speed of the bearing and the cage skidding ratio were calculated (Figure S3c). The experimental results indicate that the rotational frequency in the NSE-TBS output signal exhibits an extremely high linear correlation with the motor speed, with a linear fitting coefficient of $R^2 = 1.0000$. In addition, the maximum cage skidding ratio observed at 100 rpm is only 0.29%. These results demonstrate that lubrication has a minimal effect on the output performance of the NSE-TBS, and the sensor can still achieve high-precision rotational speed tracking and skidding monitoring under complex conditions such as lubrication.

Note5: Structural Parameters and Hyperparameter Configuration of the 1D-ViT Model.

The architectural parameters of the 1D-ViT model and the training hyperparameters are defined in Tables S3 and S4, respectively.

Table S3: Architecture of the 1D-ViT Model

Layer	Input Shape	Output Shape
Input	(1,2048)	(1,2048)
Patch Embedding	(1,2048)	(64,128)
Class Token	(64,128)	(65,128)
Pos. Encoding	(65,128)	(65,128)
Transformer Block 1	(65,128)	(65,128)
Transformer Block 2	(65,128)	(65,128)
LayerNorm	(65,128)	(65,128)
Classification Head	128	12 (10)

Table S4: Definition of 1D-ViT Model Hyperparameters

Hyper-parameter	Value	Hyper-parameter	Value
Signal length	2048	Feed-forward-dim	256
Patch size	32	Dropout	0.1
Embedding-dim	128	Batch size	32
Attention Heads	8	Learning Rate (Adam)	0.001
Encoder Layers	2	Epochs	50

Note6: Impact of Tiling Parallelism on FPGA Hardware Performance.

The proposed hardware acceleration scheme is based on a general-purpose matrix multiplication architecture, where the computational parallelism is determined by the tiling factor TILE_SIZE, which directly corresponds to the size of the processing element (PE) array. In principle, increasing TILE_SIZE can significantly improve inference speed; however, it must be carefully optimized due to the limited on-chip resources of the FPGA. In this work, the IP core was developed using Vivado HLS, and synthesis as well as bitstream generation were carried out in Vivado 2023.2. The experimental results are summarized in Table S5. When TILE_SIZE = 64, the DSP utilization (1319 units) exceeds the available resources of the ZYNQ-7045 (900 units), making hardware deployment infeasible. Although TILE_SIZE < 32 reduces resource consumption and power usage, the prolonged computation cycles lead to an increase in energy per inference (EPI). Overall, TILE_SIZE = 32 achieves the best trade-off among hardware resource utilization, inference efficiency, and energy efficiency, and is therefore selected as the optimal configuration in this study.

Table S5: Impact of Different TILE_SIZE on the Hardware Performance of the ZYNQ-7045 FPGA

TILE_SIZE	LUT	FF	DSP	Power	Inference Time	EPI
64	177230 (81%)	298628 (38%)	1319 (146%)	\	\	\
32	96094 (43.96%)	138114 (31.59%)	513 (57.00%)	4.588W	0.108ms	0.496mJ
24	78593 (35.95%)	117391 (26.85%)	386 (42.89%)	3.867W	0.231ms	0.895mJ
12	24161 (11.05%)	33706 (7.71)	167 (18.56%)	2.458W	0.496ms	1.219mJ

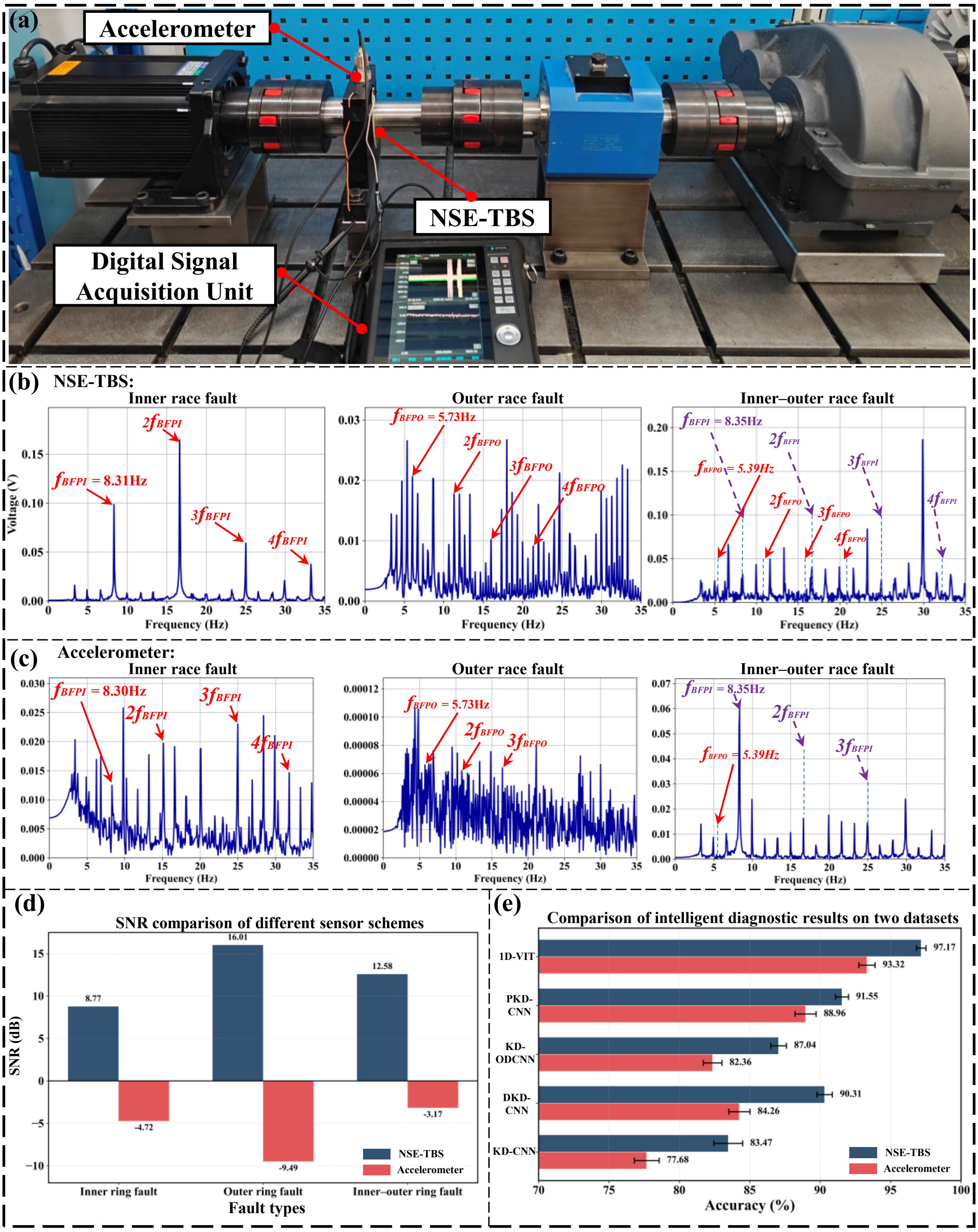

Figure S4: Performance comparison between the NSE-TBS and the Accelerometer: a) experimental setup, b) fault characteristic frequencies extracted from NSE-TBS signals, c) fault characteristic frequencies extracted from Accelerometer signals, d) SNR comparison, e) intelligent diagnostic performance comparison.

Note7: Performance Comparison between NSE-TBS and the Vibration Accelerometer.

To comprehensively evaluate the sensing performance of the NSE-TBS and a conventional vibration accelerometer, experiments were conducted on a self-built load test rig. As shown in Figure S4a, a vibration accelerometer (sensitivity: 100 mV/g; measurement range: ±50 g) was mounted directly above the bearing housing to acquire vibration signals. Four typical bearing conditions were considered, including normal condition (NC), inner race fault (IF), outer race fault (OF), and compound inner–outer race fault (IOF). The test conditions included constant rotational speeds (100 rpm and 250 rpm) and a variable speed condition (0–500 rpm with a linear acceleration over 10 s). All signals were synchronously acquired using a data acquisition system (COCO 8X), with a sampling duration of 10 s for each trial.

To evaluate the capability of the two sensors in capturing fault features, signals under the 100rpm condition were selected for comparative analysis. The theoretical fault frequencies of the inner and outer races are 9.0375 Hz and 5.9625 Hz, respectively. For the three typical fault conditions (IF, OF, and IOF), a 2–35 Hz band-pass filter was first applied to preprocess the signals, removing DC components and high-frequency environmental noise. Subsequently, the filtered signals were transformed using FFT, and the spectra of the NSE-TBS and the accelerometer are shown in Figure S4b and Figure S4c, respectively. The experimental results indicate that the fundamental fault frequencies extracted from the NSE-TBS signals are in good agreement with those obtained from the vibration accelerometer, validating its effectiveness in fault frequency detection. Under the OF condition, the spectrum obtained from the vibration accelerometer can only identify the third-order harmonic, while higher-order harmonics are obscured by noise. Under the IOF condition, its ability to identify the harmonic components associated with the outer race fault is also limited. In contrast, the NSE-TBS is capable of clearly extracting both the fundamental fault frequencies and multiple higher-order harmonic components under all three fault conditions, demonstrating superior feature resolution capability.

Furthermore, considering that the artificially introduced bearing defects are relatively small, the resulting impulsive features are easily masked by broadband background noise. To quantitatively evaluate the sensitivity of the two sensors to weak impulses, the signal-to-noise ratio (SNR) is adopted as a performance metric to characterize the contrast between fault-related energy and background noise, defined as $SNR = 10 \cdot log_{10}\left(\frac{P_{signal}}{P_{noise}}\right)$。As shown in Figure S4d, the NSE-TBS achieves higher SNR values than the vibration accelerometer under all operating conditions. For the three fault conditions, the SNR values of the accelerometer are all below 0 dB, indicating that the fault-related components are dominated by noise. In particular, under the OF condition, the SNR is as low as −9.49 dB, reflecting poor signal distinguishability.

This phenomenon can be attributed to two main factors. First, due to the low rotational speed and the small defect size, the impact energy is weak, resulting in vibration responses that are close to early-stage fault characteristics. Second, the amplitude of vibration signals is strongly dependent on the spatial relationship between the fault location and the sensor. When the defect is located far from the sensor, the impulsive signal undergoes significant structural attenuation and noise interference during propagation. In practical industrial scenarios, vibration accelerometers are typically mounted on the machine housing, which is relatively distant from the bearing, making the signals more susceptible to attenuation and environmental noise. In contrast, the NSE-TBS adopts an embedded design and is directly integrated within the bearing, enabling in situ sensing. This effectively shortens the propagation path between the monitoring point and the fault source, thereby allowing the acquisition of raw signals with a higher signal-to-noise ratio.

To further evaluate the impact of the two sensors on downstream intelligent diagnostic performance, four representative lightweight CNN–FPGA acceleration schemes in the field of bearing fault diagnosis were selected for comparison. These include a knowledge distillation (KD)-based CNN model (KD-CNN) [1], a single-layer CNN trained with decoupled KD (DKD-CNN) [2], a KD-trained binarized CNN model (KD-ODCNN) [3], and a progressively KD CNN model (PKD-CNN) [4]. The experimental data were collected from both vibration signals and triboelectric signals under steady-state conditions at 100 rpm and 250 rpm, as well as under a dynamic speed condition (0–500 rpm with linear acceleration). In total, 12 datasets were constructed. The data supporting the findings of this study are available at:200231w/DATA.

To ensure the statistical reliability of the results, the training settings and model architectures of the compared models were kept consistent with those reported in the original studies, and each experiment was independently repeated 10 times under different random seeds. The intelligent diagnostic results based on the two types of sensor signals are presented in Figure S4e.

It can be observed that all compared models achieve significantly better diagnostic performance when using NSE-TBS signals than when using vibration accelerometer signals. For the DKD-CNN and KD-CNN models, the relatively shallow network depth and limited number of convolutional kernels constrain their ability to represent weak fault features embedded in noisy backgrounds. When vibration accelerometer signals are used as input, the higher background noise leads to severe feature aliasing in the feature space, thereby significantly degrading classification performance. In contrast, the high signal-to-noise ratio provided by the NSE-TBS effectively enhances the separability of fault features, enabling lightweight models to achieve higher classification accuracy despite their limited representational capacity. These results collectively demonstrate that the NSE-TBS not only enables high-precision sensing of weak fault-induced impulses, but also significantly improves the performance of downstream intelligent diagnostic models by providing high-quality input data, thereby offering strong support for the development of highly reliable online monitoring systems.

Note: Experimental equipment models.

Design of the online diagnostic system: The system consists of an FPGA (ZYNQ-7045), a 16-bit ADC (AD9269), and a signal amplifier (VK201). The electrical signals acquired by the FPGA are transmitted to a PC, where they are recorded and stored via a Qt-based host application. Experimental measurement: The electrical signals output by the NSE-TBS are recorded onto a memory card via a digital signal acquisition device (COCO 8x) and then imported into a computer for analysis. Designing the test platform: The experimental test rig consists of a drive motor (130BYG3500-N, PFDE, China), a torque sensor (WTQ-2053), coupling components, a bearing housing, a deep-groove ball bearing (6208, SKF), a two-stage reducer, and a magnetic particle brake (CZ-20).

Reference

[1] M. Ji, G. Peng, S. Li, F. Cheng, Z. Chen, Z. Li, and H. Du, “A neural network compression method based on knowledge-distillation and parameter quantization for the bearing fault diagnosis,” Applied Soft Computing, vol. 127, DOI 10.1016/j.asoc.2022.109331, p. 109331, 7 2022.

[2] J.-X. Liao, S.-L. Wei, C.-L. Xie, T. Zeng, J. Sun, S. Zhang, X. Zhang, and F.-L. Fan, “BearingPGANet: a lightweight and deployable bearing fault diagnosis network via decoupled knowledge distillation and FPGA acceleration,” IEEE Transactions on Instrumentation and Measurement, vol. 73, DOI 10.1109/tim.2023.3346517, pp. 1–14, 12 2023.

[3] Z.-S. Syu and C.-H. Lee, “One-Dimensional Binary Convolutional Neural Network Accelerator design for bearing fault diagnosis,” IEEE Sensors Journal, vol. 24, DOI 10.1109/jsen.2023.3340715, no. 3, pp. 3649–3658, 12 2023.

[4] K. Zhu, X. Li, Z. Liu, J. Zhang, and Y. Wang, “An efficient fault diagnosis framework for rotating machinery combining progressive knowledge distillation and FPGA-Based acceleration,” IEEE
Transactions on Instrumentation and Measurement, vol. 75, DOI 10.1109/tim.2026.3666004, pp. 116, 1 2026.
